# Exploring the comparative genome of rice pathogen *Burkholderia plantarii*: unveiling virulence, fitness traits, and a potential type III secretion system effector

**DOI:** 10.3389/fpls.2024.1416253

**Published:** 2024-05-23

**Authors:** Mohamed Mannaa, Duyoung Lee, Hyun-Hee Lee, Gil Han, Minhee Kang, Tae-Jin Kim, Jungwook Park, Young-Su Seo

**Affiliations:** ^1^ Department of Integrated Biological Science, Pusan National University, Busan, Republic of Korea; ^2^ Institute of System Biology, Pusan National University, Busan, Republic of Korea; ^3^ Department of Plant Pathology, Faculty of Agriculture, Cairo University, Giza, Egypt; ^4^ Biotechnology Research Division, National Institute of Fisheries Science, Busan, Republic of Korea

**Keywords:** type III secretion system, secondary metabolites, genome analysis, rice pathogenic bacteria, virulence

## Abstract

This study presents a comprehensive genomic analysis of *Burkholderia plantarii*, a rice pathogen that causes blight and grain rot in seedlings. The entire genome of *B. plantarii* KACC 18964 was sequenced, followed by a comparative genomic analysis with other available genomes to gain insights into its virulence, fitness, and interactions with rice. Multiple secondary metabolite gene clusters were identified. Among these, 12 demonstrated varying similarity levels to known clusters linked to bioactive compounds, whereas eight exhibited no similarity, indicating *B. plantarii* as a source of potentially novel secondary metabolites. Notably, the genes responsible for tropolone and quorum sensing were conserved across the examined genomes. Additionally, *B. plantarii* was observed to possess three complete CRISPR systems and a range of secretion systems, exhibiting minor variations among the analyzed genomes. Genomic islands were analyzed across the four genomes, and a detailed study of the *B. plantarii* KACC 18964 genome revealed 59 unique islands. These islands were thoroughly investigated for their gene contents and potential roles in virulence. Particular attention has been devoted to the Type III secretion system (T3SS), a crucial virulence factor. An *in silico* analysis of potential T3SS effectors identified a conserved gene, *aroA*. Further mutational studies, *in planta* and *in vitro* analyses validated the association between *aroA* and virulence in rice. Overall, this study enriches our understanding of the genomic basis of *B. plantarii* pathogenicity and emphasizes the potential role of *aroA* in virulence. This understanding may guide the development of effective disease management strategies.

## 1 Introduction

The *Burkholderia* genus, a member of the *β*-proteobacteria subphylum, encompasses gram-negative bacterial species that are distinguished by their genetic versatility and adaptability to various ecological niches (O’Sullivan and Mahenthiralingam, 2005). These microorganisms are known for their high genetic plasticity and large multi-replicon genomes, which are rich in insertion sequences and genomic islands. These genomes contain a high proportion of coding regions, providing these bacteria with a substantial metabolic capacity to produce various metabolites capable of degrading environmental substances. This metabolic capacity enables their survival and fitness across a multitude of habitats (Compant et al., 2008; Seo et al., 2015; Mannaa et al., 2018).

Within the *Burkholderia* genus, three plant pathogenic species have been identified that cause diseases in rice plants: *B. glumae, B. gladioli*, and *B. plantarii*. *B. glumae* and *B. gladioli* have been extensively studied, but *B. plantarii* has received relatively little attention. *B. plantarii* was identified in Japan in 1985 and has subsequently spread to China and Korea (Azegami et al., 1987; Ra et al., 2016; Wang et al., 2016). This pathogen, once believed to affect only specific stages of rice growth, has recently been implicated in causing more severe rice diseases and has exhibited the ability to spread to other geographic locations. In addition to seedling blight, this pathogen causes grain rot, demonstrating its unique and damaging characteristics (Ra et al., 2016). This pathogen inflicts various symptoms in rice seedlings, including chlorosis, stunting, and inhibition of root growth, as well as grain rot symptoms such as grain abortion and discoloration of the hull and grain at the flowering and maturity stages (Azegami et al., 1988; Ra et al., 2016).

*B. plantarii* virulence is primarily attributed to its production of tropolone, a potent phytotoxin. Tropolone virulence in rice seedlings is facilitated by a powerful cationic metal chelating effect (Wang et al., 2013). However, the pathogenicity of *B. plantarii* might not rely solely on tropolone. Certain plant species remain symptom-free when exposed to pure tropolone, whereas inoculation with tropolone-producing *B. plantarii* triggers disease development (Iiyama et al., 1998). This indicates that other, less understood virulence factors may also play significant roles in the disease process.

One essential element among the various virulence factors implicated in bacterial infections in eukaryotic hosts is the type III secretion system (T3SS). This complex and specialized macromolecular structure, embedded in the membranes of gram-negative bacteria, serves as a protein delivery machine for translocation into eukaryotic cells (Reinhold-Hurek and Hurek, 2011; Angus et al., 2014). T3SS can be used to inject protein effectors into the host, a feature exploited by many bacterial pathogens, emphasizing the potential association between the presence of T3SS and virulence in *B. plantarii*. The T3SS is common among pathogenic bacterial strains, but it is relatively rare among plant-beneficial strains and endophytes. Consequently, T3SS is frequently considered a pathogenic marker (Angus et al., 2014; Wallner et al., 2021).

The significance of the T3SS in pathogenicity has been observed in *B. plantarii* related species. For instance, in *B. glumae*, the T3SS has been found to efficiently translocate effectors to plant cells, contributing to its virulence (Sharma et al., 2013). The T3SS of *B. gladioli* has not yet been thoroughly investigated for its role in rice pathogenesis; however, it has been implicated in mycophagy, which is promoted by the injection of a prophage-tail-like effector via the T3SS of *B. gladioli* (Swain et al., 2017). Given these observations, investigating T3SS and understanding its role and potential effectors in *B. plantarii* is critical for unraveling the mechanisms of virulence and identifying avenues for effective disease management.

The advent of high-throughput sequencing technologies has revolutionized the field of bacterial genomics and paved the way for advanced studies on pathogen biology (Goodwin et al., 2016). Comparative genomics has emerged as a potent approach to investigate the virulence, adaptation, and evolutionary dynamics of bacterial pathogens, providing vital insights into their interactions with host organisms (Land et al., 2015). Application of these techniques to *B. plantarii* has revealed unique genetic traits associated with pathogenicity and fitness, enhancing our understanding of the biology of the pathogen and its interactions with the rice host (Seo et al., 2015).

Despite advancements in our understanding, significant gaps remain regarding the genomic characteristics of *B. plantarii*, particularly those contributing to its fitness and virulence. Thus, this study aimed to conduct a thorough genomic analysis of *B. plantarii* utilizing the complete and nearly complete genomes for elements potentially linked to bacterial virulence and fitness. Our focus extended to features such as secondary metabolite production, quorum sensing (QS), tropolone biosynthesis, CRISPR systems, genomic islands, and a variety of secretion systems. A particular focus was placed on identifying potential T3SS effectors and examining the role of a specific effector gene in the virulence of *B. plantarii* in rice, along with its possible contributions to other physiological functions. Through this detailed genomic exploration, we aim to enhance our understanding of the pathogenic mechanisms employed by *B. plantarii* and establish a solid foundation for subsequent research into a critical virulence effector, which may have a close association with the T3SS.

## 2 Materials and methods

### 2.1 Bacterial strains, genomic sequencing, and data collection

Complete and nearly complete genomes of *B. plantarii* strains were downloaded from the National Center for Biotechnology Information (NCBI) database. In addition, the complete genome of *B. plantarii* KACC 18964, originally isolated from rice panicles showing blight symptoms in South Korea, has been sequenced (Ra et al., 2016). To sequence its whole genome, a pure culture of the bacterium was obtained by streaking it on Luria-Bertani (LB) agar, followed by incubation at 28°C. A single colony from the plate was transferred to fresh LB broth and incubated in a shaking incubator at 200 rpm and 28°C. Genomic DNA was extracted from this culture using a Wizard Genomic DNA Purification Kit (Promega, Madison, WI, USA) according to the manufacturer’s protocol. The quantity and purity of the DNA samples were assessed using a NanoDrop 2000 spectrophotometer (Thermo Fisher Scientific, Waltham, MA, USA).

The whole genome sequence library of genomic DNA of *B. plantarii* KACC 18964 was prepared using the SMRTbell template prep kit (Pacific Biosciences, Menlo Park, CA, USA). Single-molecule real-time (SMRT) sequencing was performed using the PacBio RS II platform (Pacific Biosciences) at Macrogen (Seoul, South Korea). To improve contig accuracy, HiSeq3000 (Illumina, San Diego, CA, USA) paired-end reads were used for sequence compensation. The hierarchical genome assembly process (HGAP) version 3 in the PacBio SMRT Analysis algorithm version 2.3.0 package was implemented for pre- and *de novo* assemblies of long reads (Chin et al., 2013). Quality control of the long reads was performed using the PreAssembler filter version 1 protocol from HGAP, and the sequences were subsequently polished with Quiver to enhance quality (Chin et al., 2013). Pilon version 1.21 was used to map the HiSeq reads to the PacBio assembly twice, improving the accuracy of the genome assembly (Chin et al., 2013). Initially, genomic features were predicted and annotated using Prokka version 1.13 (Seemann, 2014), and re-annotation was performed using the NCBI prokaryotic genome annotation pipeline based on the best-placed reference protein set and GeneMarkS+ (Besemer et al., 2001). Genomic resources of *B. plantarii* KACC 18964 have been deposited in the NCBI GenBank (GCA_030644525.1).

The genome sequence of the KACC 18964 strain was further annotated using the Rapid Annotation Subsystem Technology toolkit (Brettin et al., 2015). Subsystem categories were assigned, and the number of genes was determined using the Bacterial and Viral Bioinformatics Resource Center (https://www.bv-brc.org/). To visualize genomic features, a circular feature map was generated using the CGView server (Stothard et al., 2019). For comparative analysis, the genomic sequences of all *B. plantarii* strains were downloaded from the FTP site of the RefSeq database at NCBI (ftp://ftp.ncbi.nih.gov/genomes).

### 2.2 Analysis of comparative genomic and phylogenomic relationship among *B. plantarii* strains

The whole genome sequence of *B. plantarii* KACC 18964 was compared to that of three other *B. plantarii* strains (ATCC 43733, PG1, and ZJ171) as well as the congeneric outgroup species *B. glumae* BGR1. Average nucleotide identity (ANI) values were computed using the OrthoANIu algorithm in EzBioCloud to assess the similarity between the complete genome sequences of *B. plantarii* KACC 18964 and other strains (Yoon et al., 2017).

Whole-genome alignment and digital DNA-DNA hybridization (dDDH) values were calculated using the genome-to-genome distance calculator web server with Formula 2 (http://ggdc.dsmz.de/distcalc2.php). Additionally, the KACC 18964 genome was compared with other related genomes of *Burkholderia* type strains using the Type (Strain) Genome Server (TYGS) (Meier-Kolthoff et al., 2013). Phylogenomic pairwise comparisons were performed among a set of related *Burkholderia* genomes using the genome BLAST distance phylogeny (GBDP) approach. Accurate intergenomic distances were inferred using the ‘trimming’ algorithm and the distance formula *d5* with 100 distance replicates (Meier-Kolthoff et al., 2013; Meier-Kolthoff and Göker, 2019).

Based on these intergenomic distances, a balanced minimum evolution tree was constructed using FASTME 2.1.6.1. Branch support was inferred from 100 pseudo-bootstrap replicates (Lefort et al., 2015). To cluster genomes into species, type-based species clustering was performed using a 70% dDDH radius around each type strain (Meier-Kolthoff and Göker, 2019). The resulting tree was visualized using iTOL v5 (Letunic and Bork, 2021).

Pan-genome analysis was performed using Roary version 3.11.2 (Page et al., 2015). To generate input files for Roary, the gff format files were derived from the gbk format files of each strain, which were downloaded from the NCBI RefSeq database using a custom Python script. The CD-HIT algorithm was used in Roary (Fu et al., 2012) with default parameters, including a minimum percentage identity of 95% for blastp and a core gene threshold of 99% occurrence across the strains, to cluster orthologous gene families and calculate the pan-genome of the four *B. plantarii* strains and *B. glumae* BGR1. The Roary pipeline incorporates the PRANK aligner (Löytynoja, 2014) to create a concatenated multiple sequence alignment of the core gene families. To construct a maximum-likelihood tree from the core gene alignment, RAxML version 8 was used (Stamatakis, 2014). The best-scoring tree was generated using the GTRGAMMA model, supported by 1000 rapid bootstrap replicates. The resulting tree was visualized using iTOL v5 (Letunic and Bork, 2021). To graphically represent the distribution of the pan-genome across the strains, we utilized the roary_plots Python scripts (Galardini, 2017). These scripts used the core genome tree constructed from the RAxML tool, along with the gene presence/absence matrix obtained from Roary analysis.

### 2.3 *In silico* functional annotation and examination of QS, tropolone, and putative secondary metabolite gene clusters

Whole-genome mining was employed to identify potential functional elements. Particularly, we investigated the QS I/R system (*plaI, plaM*, and *plaR*) of *B. plantarii*, as well as the tropolone biosynthesis genes across multiple *B. plantarii* strains (ATCC 43733, KCCM 18964, PG1, and ZJ171).

To assess the potential for secondary metabolite production in *B. plantarii* KACC 18964, we used the antiSMASH tool server, which predicts secondary metabolite biosynthesis gene clusters (Blin et al., 2021). The outcomes were then visualized by mapping the predicted gene clusters onto a circular representation of the genome, and the similarity levels to known gene clusters were determined.

### 2.4 CRISPR/Cas system analysis and characterization

The CRISPR regions and CRISPR-associated Cas proteins were predicted in all *B. plantarii* and *B. glumae* BGR1 genomes using CRISPRCasFinder (Couvin et al., 2018). For this analysis, only CRISPR arrays with an evidence level > 3 were considered to avoid potential false-positive results, such as short arrays with one to three spacers. The arrays identified from CRISPRCasFinder were cross-verified with the results from CRISPRDetect version 2.4 (Biswas et al., 2016) and the corresponding NCBI genome annotations.

To characterize the consensus sequences, CRISPR repeat sequences were aligned using T-COFFEE (Notredame et al., 2000) with default parameters and presented using ESPript programs (Robert and Gouet, 2014). Each strain’s CRISPR array was grouped based on its conserved sequences, and a number was assigned to each group (e.g., CRISPR 1, CRISPR 2, and CRISPR 3). The sequence logo of the T-COFFEE-aligned CRISPR DR was generated using Weblogo (Crooks et al., 2004).

To assess the inter-strain and intra-species divergence of the spacers, CRISPRStudio was used with default parameters (Dion et al., 2018). This allowed us to concatenate, compare, and visualize the spacers predicted by CRISPRCasFinder and CRISPRDetect using HEX color codes.

### 2.5 Genomic island prediction

Genomic island prediction for *B. plantarii* was performed using two distinct tools. Initial comparative analysis utilized IslandCompare v1.0 (Bertelli et al., 2022), for cross-genome GI content exploration within the four studied *B. plantarii* to identify GIs, and antimicrobial gene determinants enabling comparative analysis in tested genomes.

Subsequent in-depth prediction and analysis of GIs within the *B. plantarii* KACC18964 genome were conducted using IslandViewer 4 (Bertelli et al., 2017), an integrated tool for the computational identification and visualization of GIs and antimicrobial resistance genes, combining IslandPath-DIMOB, and SIGI-HMM prediction algorithms to provide a comprehensive overview of potential GIs. Gene content within predicted GIs was assessed for virulence potential, focusing on toxins, effectors, transport, and secretion systems-related genes.

### 2.6 Prediction of the secretion systems in *B. plantarii* and potential effectors of the type III secretion system

The detection and visualization of different secretion systems in all *B. plantarii* genomes were performed using MacSyFinder software (Abby et al., 2014) and associated models (Abby et al., 2016), which employ hidden Markov model profiles for efficient genomic detection of bacterial secretion systems with default parameters. The secretion systems detected in the four *B. plantarii* strains were compared based on the structure of the gene clusters.

After confirming the presence of the type III secretion system, associated effector proteins were predicted from both *B. plantarii* and *B. glumae* using EffectiveDB (Eichinger et al., 2016), a comprehensive tool that integrates various algorithms to recognize T3SS signals (EffectiveT3), conserved binding sites of type III chaperones (EffectiveCCBD), eukaryotic-like domains (EffectiveELD), and subcellular targeting signals in the host (Predotar). The default parameters were used for the tools as follows: Effective T3, minimal score = 0.9999 (2.0.1 model); EffectiveELD, minimal score = 4; and Predotar, plant model. Only high-confidence predictions were included in the results. The predicted specific effector that was identified by all algorithms was considered for further analysis to explore the potential mechanisms of interaction with the plant host cells and its involvement in virulence in a rice *in planta* assay, and *in vitro* characterization of its involvement with various physiological functions in *B. plantarii*.

### 2.7 Mutant generation and *in planta* assay to evaluate the involvement of *aroA* in virulence in rice

After investigating the potential T3SS effectors, the *aroA* gene (locus tag: GIY62_15125, 1302 bp) was identified as a potential effector by all four algorithms used in the examined strains, suggesting its potential involvement in the virulence of *B. plantarii* in rice plants. To examine the potential involvement of *aroA* in *B. plantarii* KACC 18964 virulence, deletion mutant (***Δ*** *aroA*) and complemented (C*aroA*) strains were generated as previously described (Lee et al., 2019). Briefly, standard protocols (Sambrook et al., 1989) were used for DNA amplification, recombinant DNA construction, and mutant strain generation. To create ***Δ*** *aroA* strains, the upstream (L fragment) and downstream (R fragment) regions were amplified from *B. plantarii* KACC18964 gDNA using Solgent Pfu-X DNA polymerase and primers with the appropriate restriction sites (**Supplementary Table S2**). These fragments were ligated into the pK18mobsacB suicide vector after digestion with specific restriction enzymes (Schäfer et al., 1994). The recombinant plasmids were introduced into *E. coli* DH5α λpir cells, transferred to *E. coli* S17–1 λpir (donor strain), and transformed into *B. plantarii* KACC18964 (recipient strain) through conjugation (Simon et al., 1983). Deletion mutants were selected using appropriate antibiotics, and a second homologous recombination was induced by subculturing them in LB containing 30% sucrose. To generate C*aroA* strains, the complete open reading frame of *aroA* was amplified and inserted into the pBBR1MCS-2 expression vector (Kovach et al., 1995). The recombinant vectors were introduced into each mutant strain via conjugation. Polymerase chain reaction (PCR) was performed to confirm the mutant and complemented strains. The strains and plasmids used are listed in **Supplementary Table S3**.

The generated ***Δ*** *aroA*, C*aroA*, and wild-type strains were evaluated in rice using *in planta* assays. Pure cultures of the wild type and mutant strains were grown on LB agar plates at 28°C for 48 h. Single colonies were then transferred into 4 mL of LB broth and grown overnight at 28°C with shaking. Subsequently, 100 µL of the cultures were transferred into 50 mL of LB broth and grown for an additional 48 h at 28°C with shaking. Bacterial cells were harvested by centrifugation at 4000 g for 20 min. The cells were washed twice and resuspended in sterilized 10 mM MgSO_4_. The optical density of the bacterial suspensions was adjusted to OD_600_ nm = 0.8 (equivalent to approximately 10^9^ cells/mL).

Rice plants used in this experiment were cultivated under greenhouse conditions with an average temperature of 30°C during the day and 25°C at night. At the flowering stage, the rice panicles were inoculated by dipping them in 30 mL of the bacterial suspension in a glass tube for 1 min. Ten days post-inoculation (dpi), the disease severity in the rice panicles was assessed and photographed. Sterilized 10 mM MgSO_4_ buffer was used as a negative control. Disease severity evaluation was performed using a scale of disease severity ranking from 0 to 5: 0 for panicles with asymptomatic healthy grains, 1 for panicles with 0–20% symptomatic grains, 2 for panicles with 21–40% symptomatic grains, 3 for panicles with 41–60% symptomatic grains, 4 for panicles with 61–80% symptomatic grains, and 5 for panicles with 81–100% symptomatic grains. Disease severity was calculated using the following formula:


Disease severity =Σ (number of grains per rank × rank value)/total number of panicles

For the seedling assay, rice seeds underwent surface sterilization with 70% ethanol and 1% NaOCl for 5 min, followed by three rinses in sterile distilled water (SDW) and drying on sterile paper. The seeds were then immersed in the bacterial suspensions with the control group treated with 10 mM MgSO_4_. After a 3-day dark incubation at 28°C, germinated seeds were transferred to plant culture containers with sterilized Kimtech tissue and 25 mL of SDW. The growth chamber maintained a 28°C average temperature and a 12-hour day/night light cycle. Seedlings were subsequently assessed for shoot and root length and photographed to evaluate disease severity 7 days after incubation in the growth chamber.

To evaluate the impact of *aroA* on the bacterial ability to colonize rice plants, another set of seedling assays was performed under aseptic conditions. After incubation, the entire seedling was homogenized in 10 mM MgSO_4_ and serially diluted. Subsequently, 200 µL of each dilution were spread on LB agar plates containing kanamycin (50 μg/mL) to count colony-forming units (CFUs). This procedure was conducted to assess the bacterial survivability and colonization capacity in rice plant tissues between the WT and the generated mutants.

### 2.8 *In vitro* characterization of the generated mutant

To assess the effect of *aroA* deletion on the bacterial growth, oxidative stress tolerance, extracellular protease activity, and motility, several *in vitro* assays have been conducted. The growth rate of the generated mutant *ΔaroA* and the complemented strain C*aroA* was compared to that of the wild-type. Briefly, overnight cultures of each bacterial strain were diluted to a standardized OD_600_ of 0.05 in fresh LB medium, incubated at 28°C with shaking at 200 rpm, and growth rates were evaluated by measuring the OD_600_ every 6 h.

For the motility assay, strains were grown each in LB broth to an OD_600_ of 0.8 to standardize the cell density before inoculation. For the assessment of swarming and swimming motility, LB agar plates with 0.5% and 0.3% agar concentrations were prepared, respectively. From each bacterial culture, 3 µl were transferred to the center of the respective agar plates. Plates were incubated at 28°C for 48 h to allow for bacterial motility. The surface area covered by bacterial growth was measured using ImageJ software. The analysis quantified the area to determine the extent of swarming and swimming behaviors. Comparisons of motility across the three strains were made using LSD test to determine significance, with *P* < 0.05 considered significant difference.

Tolerance to oxidative stress was assessed using disk diffusion assays (Lee et al., 2019). LB agar plates were uniformly seeded with each bacterial strain. Sterile paper disks, saturated with specific concentrations of H_2_O_2_ (7%, and 28%), were placed at the center of the agar plates. The plates were then incubated for 24 h at 28°C, after which zones of inhibition were observed to evaluate the bacterial tolerance to oxidative stress.

Extracellular protease activity was determined using LB agar plates enhanced with 2% skim milk (Mannaa et al., 2023). Wells were formed in the agar, into which the bacterial strains were inoculated. After incubation, the presence of clear zones surrounding the wells indicated protease activity, serving as a measure of the ability to degrade extracellular proteins.

### 2.9 Bioinformatic and statistical analyses

Bioinformatic analyses were conducted as follows unless stated otherwise. The gene loci were graphically represented with colored arrows using EasyFig version 2.1 (Sullivan et al., 2011). Phylogenetic trees were constructed using the neighbor-joining method with the Maximum Composite Likelihood model and 1000 bootstrap replicates unless otherwise indicated. Heatmaps were visualized using the heatmap.2 function of the ggplots package in R (https://CRAN.R-project.org/package=gplots). Circular maps were generated using CGview (Stothard et al., 2019). Data analysis for the plant, and *in vitro* assays was conducted using Statistical Analysis Systems software (SAS Institute, Cary, NC, USA). The General Linear Model procedure in SAS was used to perform an analysis of variance. Differences between the means were evaluated using the least significant difference test, and the level of statistical significance was set at *P* < 0.05.

## 3 Results

### 3.1 Genomic features and annotation of *B. plantarii* KACC18964

**Figure 1:**
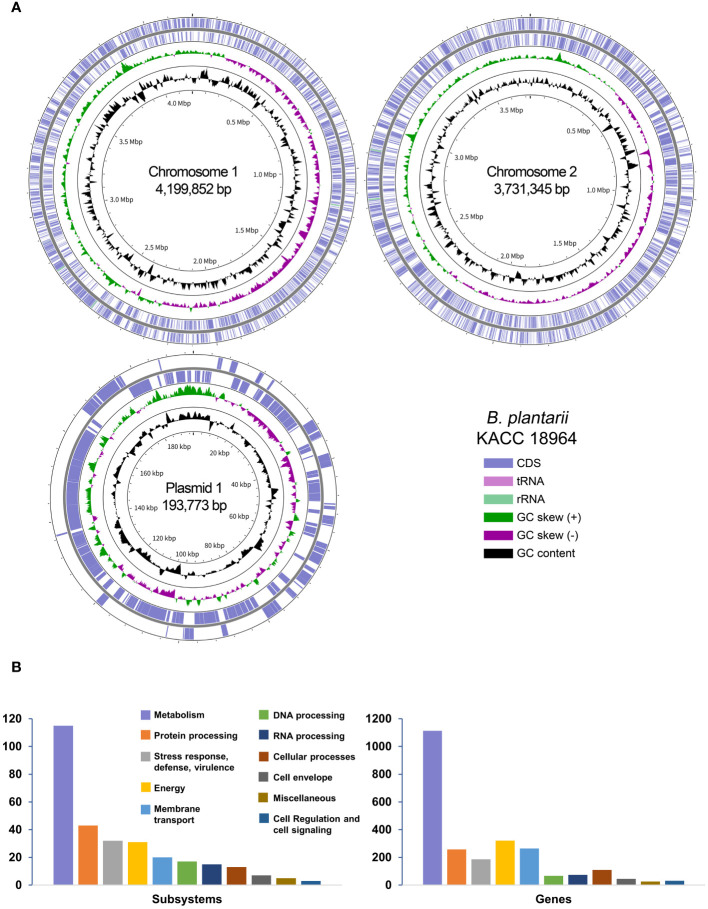
**(A)** A Circular representation of the complete genome of *Burkholderia plantarii* strain KACC 18964. The key in the bottom-right corner illustrates the individual circles in a top-down outermost to innermost direction. **(B)** The subsystem categories of the *B. plantarii* KACC 18964 genome showing the distribution of the numbers of detected subsystems and genes with assigned functions.

The complete genome of *B. plantarii* KACC 18964 was successfully assembled into two chromosomes and one plasmid, totaling 8,124,970 bp with a G+C content of 68.66%. **Table 1** provides an overview of the essential genomic features, and **Figure 1A** presents a circular view of both chromosomes and the plasmid, highlighting the coding DNA sequences (CDS), transfer RNAs (tRNAs), ribosomal RNAs (rRNAs), GC content, and positive/negative GC skew.

**Table 1:** Genomic features of *Burkholderia plantarii* KACC 18964.

Feature	Chromosome 1	Chromosome 2	Plasmid 1
Size (bp)	4,199,852	3,731,345	193,773
Genome G+C content (%)	68.38	69.30	62.27
Number of genes	3796	3123	184
Number of CDSs (with protein)	3662	3000	162
Number of pseudogenes	64	108	21
Number of RNA genes (16S/5S/23S)	3/3/3	2/2/2	0
Number of tRNA genes	57	9	1
Number of ncRNAs	4	0	0

Through RAST analysis of the *B. plantarii* KACC 18964 genome, 7,709 CDSs were identified. Among these, 5,998 CDSs (77%) were not assigned to any specific subsystem, whereas 1,711 CDSs (23%) were assigned to various subsystems. The majority of the genes were classified under metabolism (45% of assigned genes), membrane transport (13% of assigned genes), energy (11% of assigned genes), protein processing (10% of assigned genes), and stress response, defense, and virulence (7% of assigned genes). A detailed breakdown of the identified subsystem categories and the distribution of the assigned genes within these subsystems is shown in **Figure 1B**.

### 3.2 Comparative genomic analysis among *B. plantarii* strains

The complete genome sequence of *B. plantarii* KACC18964 was compared with that of other *B. plantarii* strains (ATCC 43733, KCCM 18964, PG1, and ZJ171) and *B. glumae* BGR1 obtained from the NCBI database. **Table 2** provides detailed information on the genomes used for comparison, including accession numbers and basic features.

**Table 2:** Genomes from related species used for comparison in this study.

Species	Strain	Source	Assembly	Level	Size (Mb)	GC%	Proteins
*B. plantarii*	KACC 18964	Rice	GCA_030644525.1^a^	Complete	8.12	68.66	6824
*B. plantarii*	ATCC 43733	Rice	GCA_001411805.1	Complete	8.08	68.58	6715
*B. plantarii*	PG1	Rice	GCA_000835205.1	Complete	7.90	68.78	6566
*B. plantarii*	ZJ171	Rice	GCA_001755835.1	Scaffold	8.02	68.50	6751
*B. glumae*	BGR1	Rice	GCA_000022645.2	Complete	7.28	67.93	6235

a The study strain

**Figure 2:**
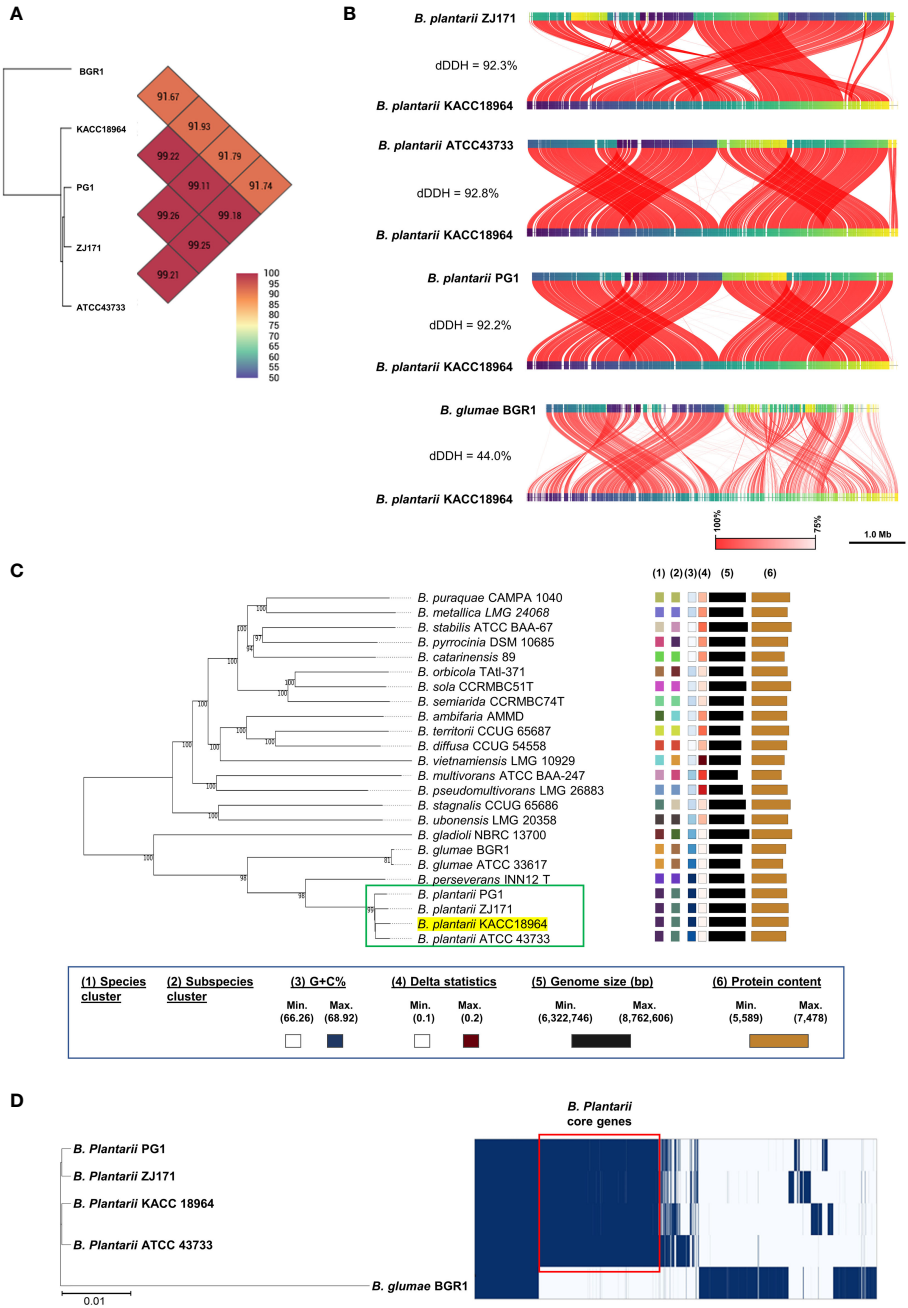
Comparative genomic analysis of *B. plantarii* KACC 18964 with other *B. plantarii* strains (ATCC 43733, PG1, and ZJ171) and *B. glumae* BGR1 as a congeneric control for differentiation. **(A)** Pairwise comparison of average nucleotide identity (ANI) among KACC 18964, the other *B. plantarii* strains (ATCC 43733, PG1, and ZJ171), and *B. glumae* BGR1. **(B)** Whole genome alignment illustrating the differences in digital DNA-DNA hybridization (dDDH) among KACC 18964, the other *B. plantarii* strains (ATCC 43733, PG1, and ZJ171), and *B. glumae* BGR1. The ANI and dDDH% values were found to be consistent with the proposed and widely accepted species boundary thresholds of 95–96% and 70%, respectively. **(C)** Phylogenomic tree constructed using TYGS (https://tygs.dsmz.de/), inferred with FastME 2.1.6.1, based on genome GBDP distances calculated from the whole genome sequences of *B. plantarii*-related species. The branch lengths are scaled using the GBDP formula *d5*, with an average branch support of 99.8%. The tree is rooted at the midpoint. **(D)** Matrix of presence/absence genes generated using Roary software, accompanied by the maximum-likelihood phylogenetic tree of *B. plantarii* strains (KCCM 18964, ATCC 43733, PG1, and ZJ171) and *B. glumae* BGR1. Blue bars indicate the presence of specific genes across the panel, with genes shared among all *B. plantarii* strains outlined in red (core genome).

*In silico* analyses were performed to determine the relatedness of the sequenced KACC 18964 genome to the other *B. plantarii* and *B. glumae* genomes in terms of ANI and dDDH. The results demonstrated that the ANI and dDDH values fell within species boundaries, consistent with the species identification (**Figures 2A, B**). To further explore the phylogenetic relationships, a phylogenomic tree was constructed using GBDP based on the whole-genome sequences of *B. plantarii* strains and other related species within the *Burkholderia* genus. The resulting tree confirmed the taxonomic positions and grouped KACC 18964 with other strains of *B. plantarii* (**Figure 2C**).

A schematic representation of the pan-genome analysis findings of the four *B. plantarii* strains and *B. glumae* BGR1 revealed the presence or absence of genes in the core and accessory genomes (**Figure 2D**). Furthermore, the maximum likelihood phylogenetic tree constructed using the core and accessory genomes of the examined strains revealed the formation of a distinct cluster for *B. plantarii* strains, separate from the congeneric *B. glumae* BGR1. Specifically, strain KACC 18964 clustered with strain ATCC 43733, whereas strain PG1 clustered with strain ZJ171 (**Figure 2D**).

### 3.3 Virulence and fitness-related features in the genome of *B. plantarii*

#### 3.3.1 Putative secondary metabolite gene clusters

**Figure 3:**
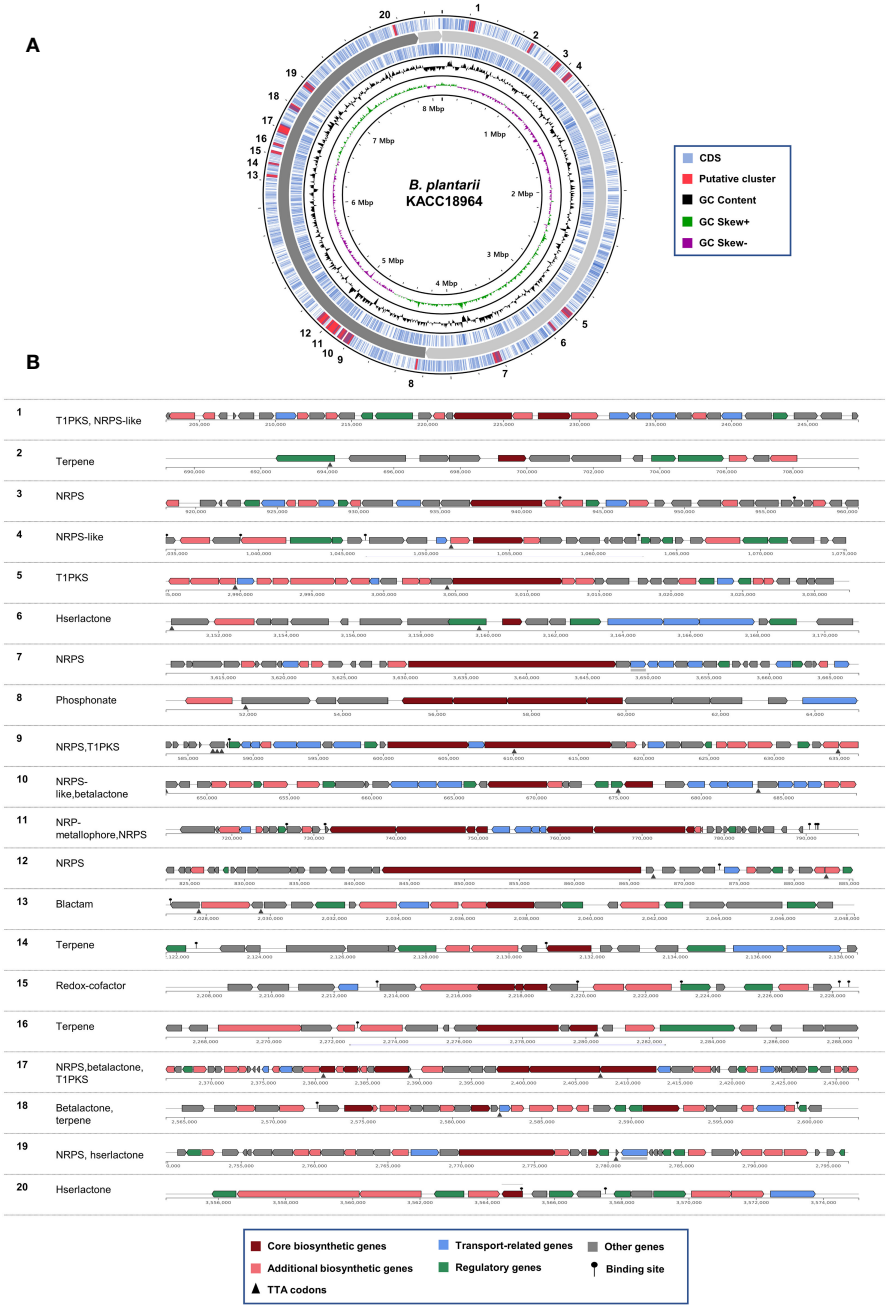
Predicted secondary metabolite gene clusters in *B. plantarii* KACC Identified by antiSMASH (version 7). **(A)** A circular representation of the whole genome indicating the locations of the detected secondary metabolite gene clusters. **(B)** Organization of gene clusters and the predicted classes of secondary metabolites. The secondary metabolite types are abbreviated as follows: T1PKS, Type I PKS (Polyketide synthase); NRPS-like, Non-ribosomal peptide synthetase-like fragment; NRPS, Non-ribosomal peptide synthetase; hserlactone, Homoserine lactone; beta lactone, Beta-lactone containing protease inhibitor; NRP-metallophore, Non-ribosomal peptide metallophores; lactam, β-lactam; redox-cofactor, Redox-cofactors such as PQQ (NC_021985:1458906–1494876).

The genome of *B. plantarii* KACC 18964 sequenced in this study served as a representative for investigating putative secondary metabolite gene clusters using antiSMASH. The analysis revealed 20 different gene clusters associated with putative secondary metabolites. Seven gene clusters were identified on chromosome I: (1) type I polyketide synthase (PKS) and non-ribosomal peptide synthetase (NRPS)-like fragment, (2) terpene, (3) NRPS, (4) NRPS-like fragment, (5) type I PKS, (6) homoserine lactone, and (7) NRPS. On chromosome II, 13 gene clusters were detected, including (1) phosphonate, (2) NRPS, (3) type I PKS, (4) NRPS-like fragment and *β*-lactone containing protease inhibitor, (5) non-ribosomal peptide metallophores and NRPS, (6) NRPS, (7) *β*-lactam, (8) terpene, (9) redox-cofactors such as PQQ, (10) terpene, (11) NRPS, *β*-lactone containing protease inhibitor, and type I PKS, (12) *β*-lactone containing protease inhibitor and terpene, and (13) NRPS and homoserine lactone. **Figure 3A** demonstrates the positions of the secondary metabolite gene clusters detected in the *B. plantarii* KACC 18964 genome. **Figure 3B** shows the representations of the gene clusters and highlights the main functions of the genes involved.

Among the identified gene clusters, 12 displayed varying degrees of similarity to known secondary metabolite clusters, whereas eight did not exhibit any similarity to known clusters (**Supplementary Table S1**). Notably, clusters 11 and 12 showed 100% similarity to Plantaribactin clusters found in other *B. plantarii* strains (Hermenau et al., 2019), and Rhizomide A/Rhizomide B/Rhizomide C clusters from *Paraburkholderia rhizoxinica* (Wang et al., 2018), respectively. Cluster 10 exhibited 62% similarity with fragin gene clusters in *Burkholderia cenocepacia* (Jenul et al., 2018). Furthermore, cluster 9 showed 41% similarity to Yersiniabactin from *Pseudomonas syringae* pv. tomato (Jones et al., 2007). Additional similarities were observed, with cluster 18 sharing 33% similarity to Barbamide from *Lyngbya majuscule* (Chang et al., 2002), cluster 5 exhibiting 25% similarity to capsular polysaccharide from *Mannheimia haemolytica* (Lo et al., 2001), cluster 17 displaying 20% similarity to Bacillomycin D from *Bacillus velezensis* (Koumoutsi et al., 2004), and cluster 13 showing 16% similarity to 1-carbapen-2-em-3-carboxylic acid from *Pectobacterium carotovorum* (McGowan et al., 1997), cluster 15 sharing 13% similarity to Lankacidin C from *Streptomyces rochei* (Suwa et al., 2000), cluster 19 exhibiting 9% similarity to Bactobolin from *Burkholderia thailandensis* (Carr et al., 2011), cluster 8 showing 6% similarity to phosphinothricin tripeptide from *Streptomyces viridochromogenes* (Schwartz et al., 2004), and cluster 4 displaying 3% similarity to Pyoverdine DC3000 from *P. syringae pv. tomato* (Ravel and Cornelis, 2003).

#### 3.3.2 QS and tropolone genes within *B. plantarii* strains

**Figure 4:**
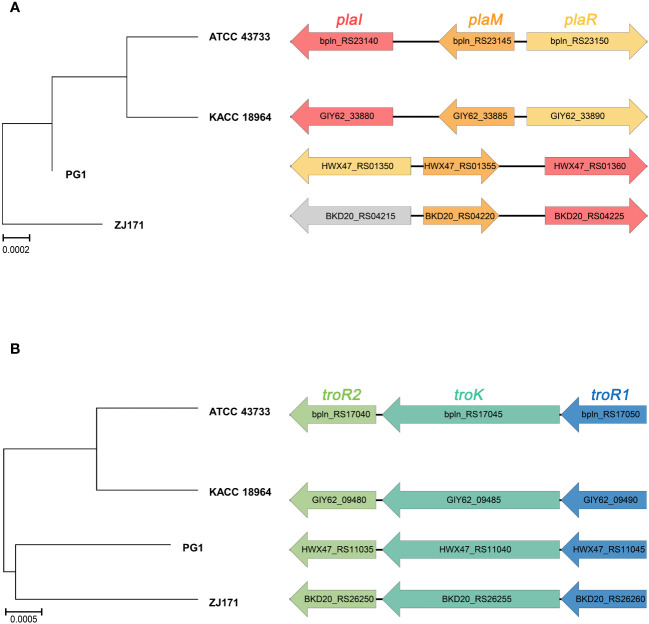
Comparison of gene cluster organization and phylogenetic tree based on sequence relatedness of **(A)** quorum sensing system and **(B)** tropolone biosynthesis genes among *Burkholderia plantarii* strains (ATCC 43733, KCCM 18964, PG1, and ZJ171).

The genomes of *B. plantarii* strains (ATCC 43733, KCCM 18964, PG1, and ZJ171) were examined to identify genes associated with the QS system. All four strains were found to have the *plaI, plaM*, and *plaR* genes involved in QS. Comparison of the organization of these genes revealed that strains ATCC 43733 and KCCM 18964 clustered together in the phylogenetic analysis, indicating their similarity, whereas strains PG1 and ZJ171 formed a separate cluster (**Figure 4A**).

Furthermore, the regulatory genes associated with tropolone production, namely *troR1*, *troR2*, and *troK*, were identified in all four *B. plantarii* strains, exhibiting a high level of similarity. In the phylogenetic analysis, strains ATCC 43733 and KCCM 18964 displayed greater similarity and clustered together, whereas strains PG1 and ZJ171 were similar and formed a distinct cluster (**Figure 4B**).

These findings imply the common presence of the QS system and tropolone biosynthesis genes among the studied *B. plantarii* strains. The clustering patterns observed in the phylogenetic analysis highlighted genetic similarities and potential evolutionary relationships between the strains.

#### 3.3.3 Prediction of CRISPR loci and *Cas* genes in *B. plantarii*

**Figure 5:**
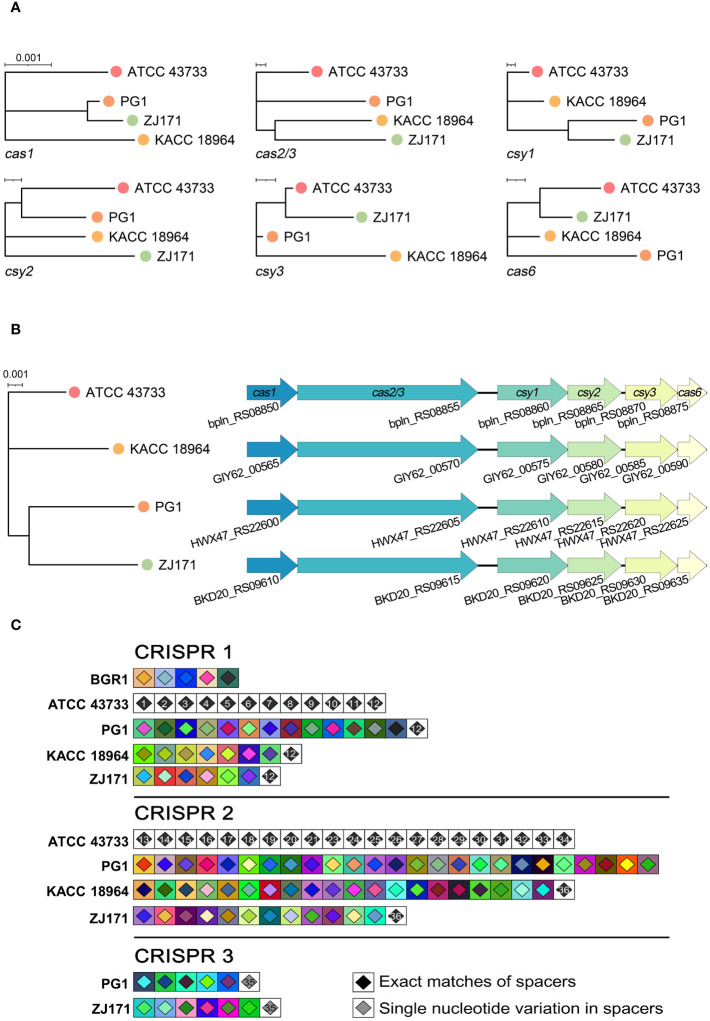
Comparative analysis of *Cas* genes and CRISPR spacers within the genomes of *B. plantarii* strains (ATCC 43733, KCCM 18964, PG1, and ZJ171). **(A)** Phylogenetic analysis based on sequence relatedness of the six detected *Cas* genes within the CRISPR systems. **(B)** Organization of *Cas* genes and phylogenetic analysis of the concatenated *Cas* genes in *B. plantarii* strains. **(C)** CRISPRStudio alignment of the arrays for three CRISPR systems, illustrating the schematic representation of spacer genes within the genomes of *B. plantarii* strains and *B. glumae* BGR1. The analysis reveals the absence of shared spacers between *B. glumae* BGR1 and *B. plantarii* strains, while all the spacers of CRISPR system 1 are common among the *B. plantarii* strains.

The classification of CRISPR systems primarily relies on the sequences of *Cas* genes, direct repeats within CRISPR arrays, and the organization of Cas operons. Within the genomes of the four *B. plantarii* strains (ATCC 43733, KCCM 18964, PG1, and ZJ171), six *Cas* genes were identified. Phylogenetic analysis based on the sequence relatedness of these *Cas* genes within the CRISPR system revealed distinct clustering patterns (**Figure 5A**). The organization of *Cas* genes and phylogenetic analysis of concatenated *Cas* genes in *B. plantarii* strains are presented in **Figure 5B**.

**Figure 5C** illustrates the CRISPRStudio alignment of arrays for the three CRISPR systems, depicting a schematic representation of spacer genes within the genomes of *the B. plantarii* strains and *B. glumae* BGR1. All four *B. plantarii* strains have CRISPR 1 and 2 spacers, with PG1 and ZJ171 also having CRISPR 3 spacers. The analysis further indicated the absence of shared spacers between the *B. glumae* BGR1 and *B. plantarii* strains. Notably, all spacers of CRISPR system 1 were common among the *B. plantarii* strains.

**Figure 6:**
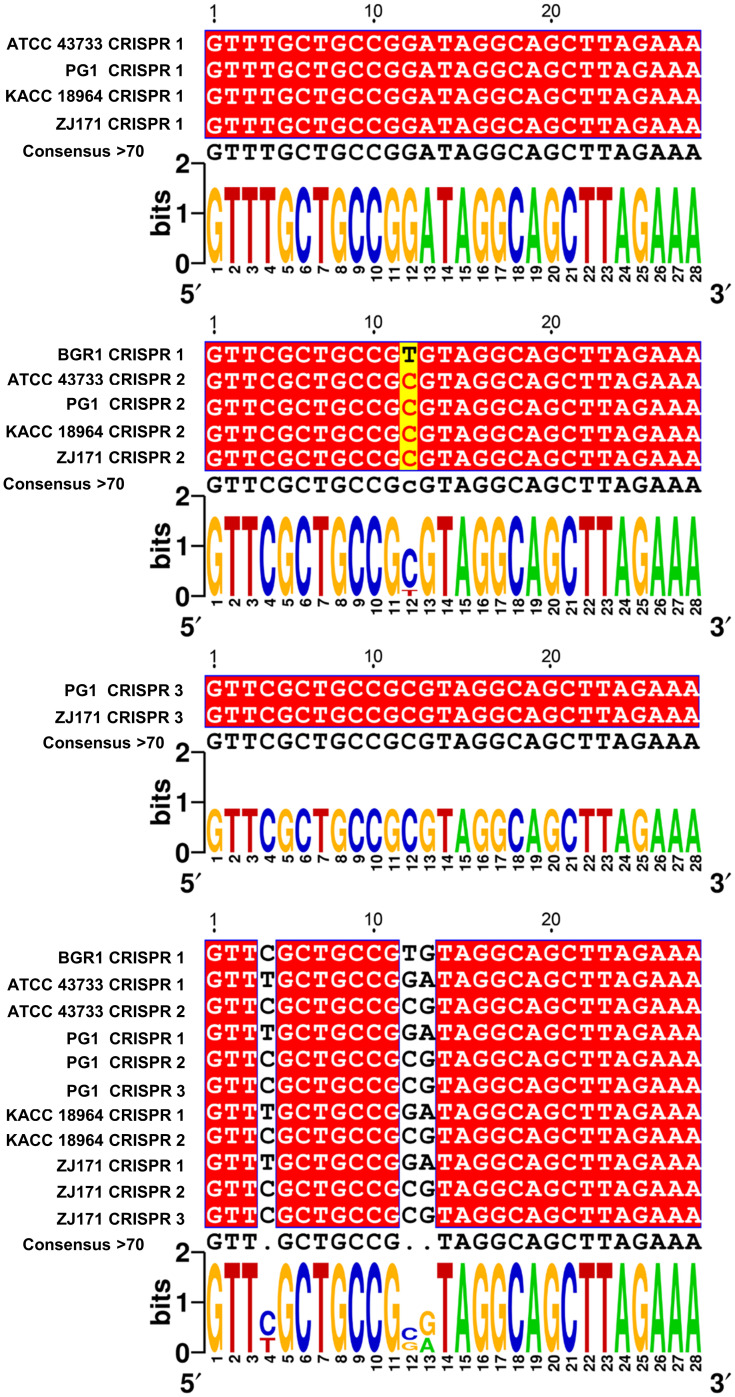
Multiple alignment of CRISPR 1–3 repeats in *B. plantarii* strains (ATCC 43733, KCCM 18964, PG1, and ZJ171) and *B. glumae* BGR1, and Weblogo analysis highlighting sequence variations and conservation among the examined strains. Multiple CRISPR systems were identified in *B. plantarii* strains, with most strains having two systems. However, strains PG1 and ZJ171 exhibited three CRISPR systems. In *B. glumae* BGR1, CRISPR 1 system repeats were detected, and their sequence showed a high similarity to CRISPR 2 in *B. plantarii*. Notably, a discrepancy was observed at the 12th sequence position, where T was present instead of C.

To explore sequence variations and conservation, multiple alignments of CRISPR repeats 1–3 were performed in *B. plantarii* strains (ATCC 43733, KCCM 18964, PG1, and ZJ171) and *B. glumae* BGR1 (**Figure 6**). Multiple CRISPR systems were identified in *the B. plantarii* strains, with most strains possessing two systems, whereas strains PG1 and ZJ171 had three systems. In *B. glumae* BGR1, CRISPR 1 system repeats were detected, showing high similarity to CRISPR 2 in *B. plantarii*. Notably, a discrepancy was observed at the 12th position, where T was present instead of C. Sequence variations and conservation among the tested strains were further analyzed using Weblogo (**Figure 6**).

#### 3.3.4 Genomic islands predicted in *B. plantarii*

**Figure 7:**
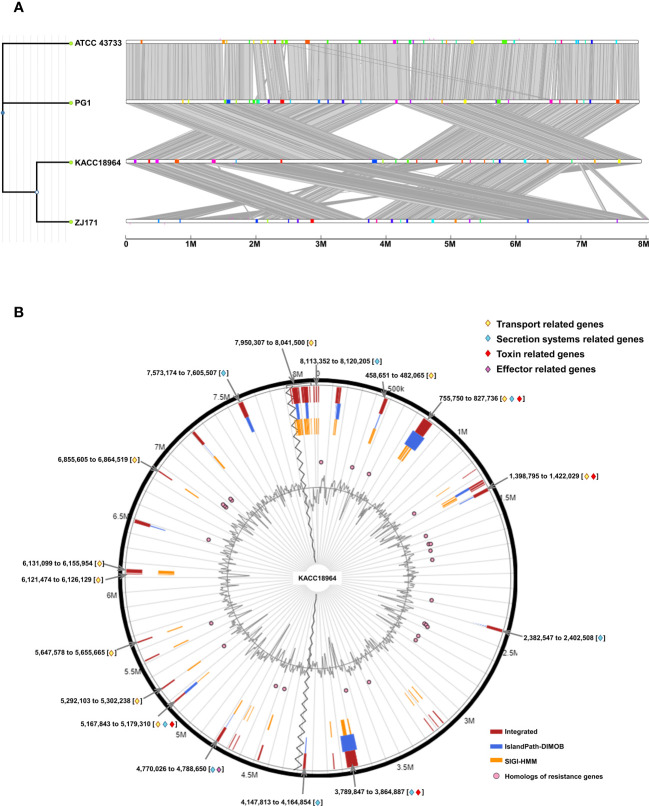
**(A)** Comparative visualization of the four *Burkholderia plantarii* genomes, generated using IslandCompare, highlights the genomic islands (GIs) and antimicrobial gene determinants. A phylogenetic tree, positioned on the upper left, elucidates the evolutionary relationships among the strains. Genomic islands (GIs) are represented as color-coded blocks aligned along the linear genome maps, **(B)**. Distribution of predicted GIs in the *B. plantarii* KACC18964 genome, as analyzed by IslandViewer 4. The circular map marks the GIs with a color-coding scheme that reflects various predictive criteria. The figure captures the genomic complexity and identifies potential hotspots for gene exchange that could influence the bacterial pathogenicity and environmental adaptation. Regions associated with potential features relevant to bacterial virulence and fitness are notably highlighted, including those containing genes related to transport, toxins, and secretion. Labels indicating the start and end points of the GIs facilitate an understanding of their genomic context.

The comparative genomic analysis using IslandCompare v1.0 revealed a complex network of GIs across the four genomes of *B. plantarii*. The phylogenetic tree elucidates the evolutionary relationships among the strains, while the linear genome maps display the GIs as color-coded blocks. Homologous regions across the genomes indicate the shared evolutionary history and potential for horizontal gene transfer (**Figure 7A**).

IslandViewer 4 analysis revealed 59 unique GIs within the genome of *B. plantarii* KACC18964 (**Figure 7B**). These islands are marked with a color-coding scheme reflecting different predictive criteria, providing insights into the genomic complexity and identifying potential hotspots for gene exchange. The GIs were evaluated for their gene content, revealing clusters of genes potentially involved in virulence, including toxins, effectors, transport, and secretion systems-related genes, indicative of bacterial environmental adaptability and pathogenicity. All the gene contents of the predicted genomic islands, specifying their start and end points, and gene annotations are listed (**Supplementary Table S4**).

In addition to the annotated GIs, the genomic landscape was predominantly characterized by genes encoding “hypothetical proteins” (247 instances), underscoring the vast unexplored genetic content within these GIs.

#### 3.3.5 Secretion systems and related genes in *B. plantarii*

**Figure 8:**
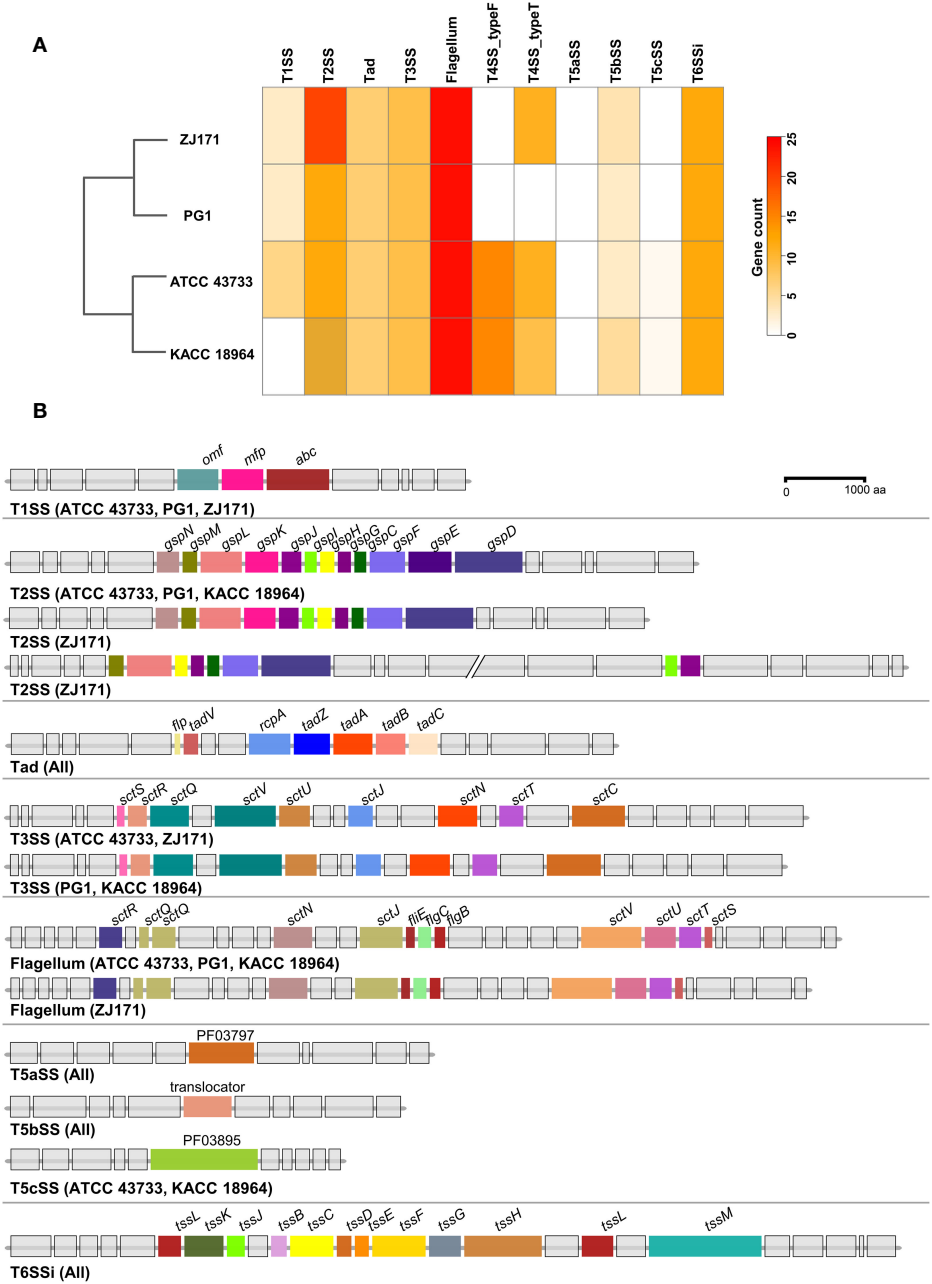
Comparison of secretion system gene clusters within the genomes of *B. plantarii* strains (ATCC 43733, KCCM 18964, PG1, and ZJ171). **(A)** A heatmap showing the number of genes identified within the detected secretion systems. **(B)** Comparative analysis of gene clusters from different detected secretion systems among *B. plantarii* strains. The type I secretion system was present in all four strains. Strain ZJ171 exhibited an additional Type II secretion system cluster. The type III secretion system showed slight variations between ATCC 43733 and ZJ171, as well as KCCM 18964 and PG1. The type IV secretion system differed among the strains, and PG1 lacked this gene cluster. The type VI secretion system exhibited the same structure and configuration across all four tested strains.

A comparative analysis of the predicted secretion systems among the four strains of *B. plantarii* (ATCC 43733, KCCM 18964, PG1, and ZJ171) revealed similarities in the types of secretion systems with minor variations. Strain KACC 18964 lacked the Type I secretion system (T1SS), which was present in the other three strains. The number of genes in the Type II secretion system (T2SS) was higher in ZJ171 than in the other strains. The Type IV secretion system, type F (T4SS_typeF) was present in KACC 18964 and ATCC 43733 but not in ZJ171 and PG1. T4SS_typeT was absent in PG1 but present in the other three strains. All strains shared the presence of Tad, T3SS, Type Vb secretion system (T5SSb), and Type VI secretion system (T6SSi). A comparison of the number of genes in each strain and phylogenetic analysis based on gene count is shown in **Figure 8A**.

The structures of the secretion system gene clusters in the four *B. plantarii* strains were compared and demonstrated in a schematic representation of the gene clusters in **Figure 8B**. The T1SS was identical in strains ATCC 43733, PG1, and ZJ171 but absent in KACC 18964. The T2SS was conserved in ATCC 43733, PG1, and KACC 18964, while in ZJ171, slight differences were observed in the identification of two clusters. Tad was conserved in the same structure in all strains. T3SS was identified in all strains, and although it was identical in ATCC 43733 and ZJ171, minor variations were observed compared with the T3SS in PG1 and KACC 18964. The flagellum gene cluster was highly conserved in ATCC 43733, PG1, and KACC 18964, with slight variation from the gene cluster in ZJ171. T5SS and T6SSi were identical in all the strains (**Figure 8B**).

#### 3.3.6 Potential effectors of T3SS in *B. plantarii*

**Figure 9:**
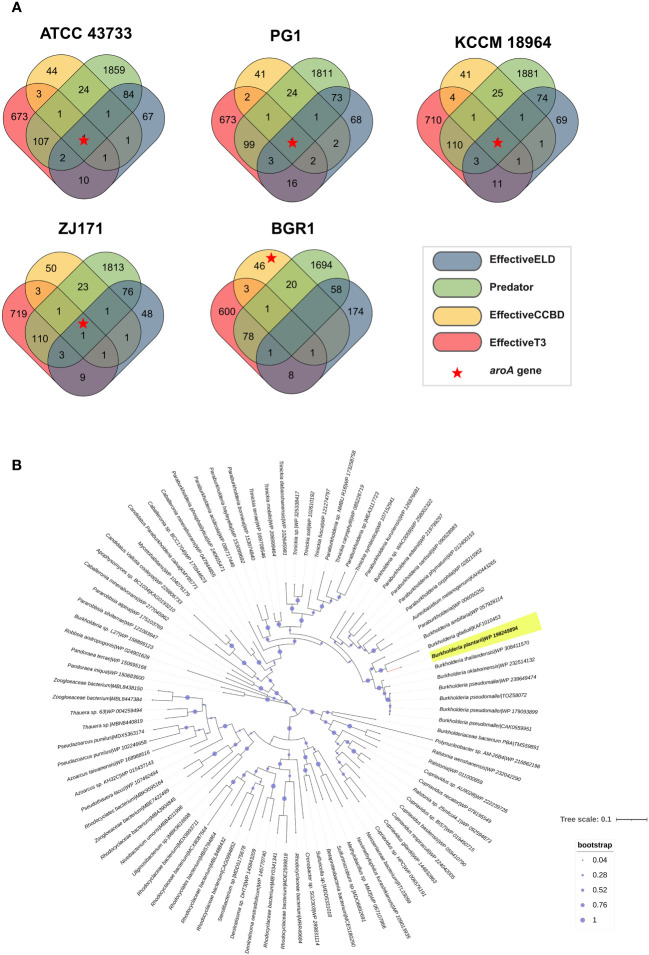
Analysis of effectors from the type III secretion system in *B. plantarii* strains (ATCC 43733, KCCM 18964, PG1, and ZJ171). and *B. glumae* BGR1. **(A)** Venn diagram demonstrating the analysis of effector genes using four different system criteria (EffectiveELD, Predator, EffectiveCCBD, and EffectiveT3). The number of detected effector genes is shown, with only one gene (highlighted as a red star) found in all analyses. This gene was also detected in *B. glumae* using the EffectiveCCBD criteria. **(B)** Phylogenetic tree generated using the maximum likelihood method with 1000 bootstrap replications, highlighting the evolutionary relationship of the aroA from *Burkholderia plantarii* (highlighted in yellow) against a backdrop of homologous sequences. Sequences were retrieved following a BLASTp search against the clustered non-redundant (nr) protein database of the NCBI, where each sequence is clustered at 90% identity and coverage. The representative sequence for each cluster is indicated, providing a compact result with increased taxonomic depth. The bootstrap values are represented by the size of the nodes, with larger nodes indicating higher support for the clade. The tree scale indicates the number of substitutions per site.

Potential effector genes were predicted using four different algorithms, namely EffectiveELD, Predator, EffectiveCCBD, and EffectiveT3, in *B. plantarii* strains (ATCC 43733, KCCM 18964, PG1, and ZJ171) and *B. glumae* BGR1. The inclusion of *B. glumae* BGR1 in the analysis was justified by its possession of a T3SS. The prediction results were visualized using a Venn diagram presenting the number of potential effectors identified (**Figure 9A**). Notably, a single gene was predicted using all four algorithms in all *B. plantarii* strains, and the same gene was detected in the *B. glumae* strain using the EffectiveCCBD algorithm. Consequently, this gene, which encodes the potential effector protein *aroA*, is suspected to function as a T3SS effector.

To provide a broader evolutionary context and underscore the ubiquity of the *aroA* gene, a phylogenetic analysis was performed. The sequences for this analysis were retrieved from a BLASTp search against the NCBI’s clustered non-redundant (nr) protein database, where sequences are clustered at 90% identity and coverage. The resulting phylogenetic tree, as depicted in **Figure 9B**, employs the maximum likelihood method with 1000 bootstrap replications. It distinctly positions the *aroA* gene of *B. plantarii* among a backdrop of homologous sequences from related organisms, highlighting its evolutionary conservation. This analysis does not directly confirm the effector functionality of *aroA* but provides essential insights into its conservation and potential roles within these bacterial species.

### 3.4 The involvement of *aroA* in *B. plantarii* virulence in rice

**Figure 10:**
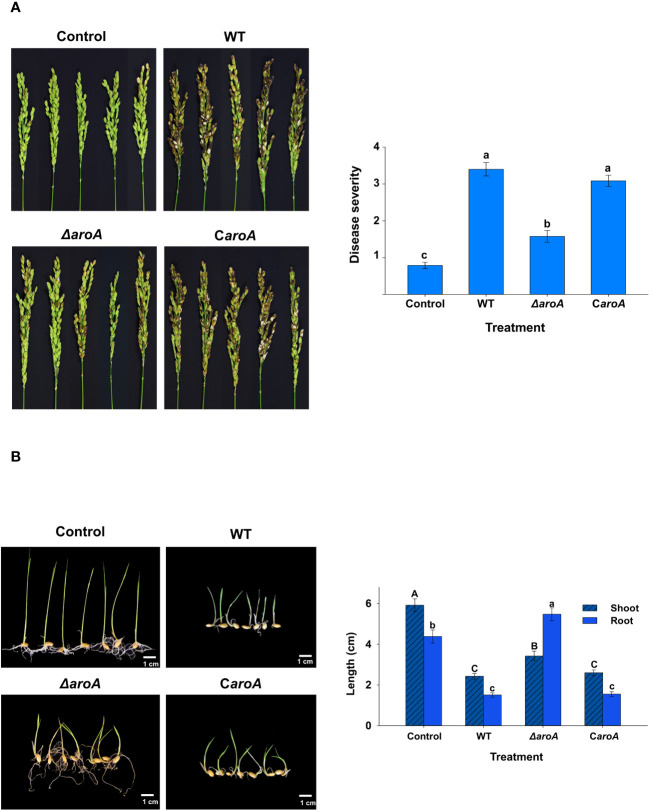
*In planta* assays for evaluation of the disease severity between the wild-type *B. plantarii* KACC18964, the mutant (*ΔaroA*), and the complemented strain (C*aroA*) **(A)** Disease severity in rice plants 10 d post-inoculation. Photographs depicting the variation in blight symptoms between the treatments. The bar graph on the right side illustrating the disease severity results in inoculated rice plants. Different letters on the error bars (Standard error, *n* = 5) indicate significant differences between the treatments, according to the least significant difference test at *P* < 0.05. A sterilized 1 mM MgSO_4_ solution was used as the negative control. **(B)** Rice seedling growth and disease severity post-inoculation. Photographs displaying the seedlings post-treatment with respective bacterial strains or control. The bar graph on the right side are showing the quantified shoot and root lengths. Seedlings treated with the *ΔaroA* deletion mutant exhibit less disease severity and greater shoot and root growth compared to those inoculated with the wild type, whereas complementation of the *aroA* mutant restores virulence to levels comparable to the wild type. Control seedlings treated with sterilized 1 mM MgSO_4_ showed no disease symptoms nor growth impairment, indicating healthy development. Different uppercase and lowercase letters on the error bars (standard error; *n*=30), indicate significant differences according to the LSD test at *P* < 0.05 for the shoot and root length, respectively.

The results of the *in planta* assays confirmed the involvement of the *aroA* gene in *B. plantarii* KACC 18964 virulence in rice. As observed in the photographs in **Figure 10A**, blight symptoms on the rice panicles treated with the mutant *ΔaroA* showed a noticeable reduction compared to those treated with wild-type *B. plantarii* KACC 18964 and the complemented strain C*aroA*.

Disease severity was assessed in accordance with this observation. A significant (*P* < 0.05) reduction in the evaluated disease severity was observed in rice treated with the mutant *ΔaroA*, compared to treatment with the wild-type KACC 18964 and the complemented strain C*aroA*. The disease severity in rice treated with the complemented strain, C*aroA*, was not significantly different from that in wild-type-treated rice (**Figure 10A**), indicating the recovery of virulence by complementation with *aroA*. These results confirm the involvement of *aroA* deletion in the partial impairment of *B. plantarii* virulence in rice.

The outcomes of the seedling development assay were consistent with those of the rice plant assay. The *ΔaroA* mutant of *B. plantarii* showed a reduction in pathogenicity. This was evidenced by enhanced seedling growth and decreased disease severity in comparison to the wild-type strain. Control seedlings maintained healthy development and exhibited no signs of disease, as depicted in the photographs (**Figure 10B**). Quantitative analysis revealed a significant increase (*P* < 0.05) in both shoot and root lengths in seedlings treated with the *ΔaroA* mutant. In contrast, the complemented strain displayed a level of pathogenicity comparable to the wild-type strain, as indicated in (**Figure 10B**).

In regards to the colonization of rice plant tissues, the bacterial population derived from seedlings treated with the wild-type strain was equivalent to 2.86 ± 0.95 log CFU/g; *n*=3, which was slightly higher than that of both the Δ*aroA* mutant (2.85 ± 0.88 log CFU/g) and the complemented strain, C*aroA* (2.73 ± 0.88 log CFU/g). However, there were no statistically significant differences between the wild type and either the mutant or the complemented strains.

### 3.5 *In vitro* analysis of *aroA* involvement in *B. plantarii* growth, stress response, protease, and motility

**Figure 11:**
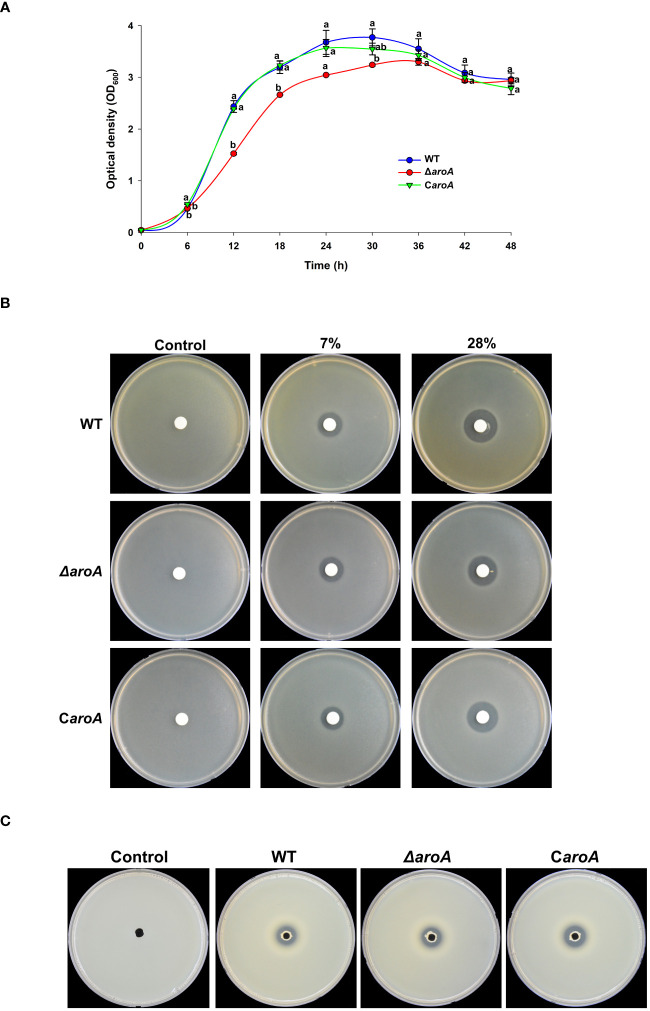
Comparative analysis of growth, oxidative stress tolerance, and protease activity in *Burkholderia plantarii* wild-type KACC18964, the mutant (Δ*aroA*), and the complemented strain (C*aroA*). **(A)** Growth curves for the WT, Δ*aroA*, and C*aroA* strains, indicating the optical density (OD_600_) measurements over 6-h intervals. Different letters above the error bars (Standard error; *n*=3) indicate statistically significant differences (*P* < 0.05) between strains at the corresponding time points. **(B)** Disk diffusion assay for H_2_O_2_ sensitivity showing zones of inhibition around disks soaked in various H_2_O_2_ concentrations. **(C)** Extracellular protease activity assays with clear proteolysis zones on LB agar plates containing 2% skim milk, indicating protease secretion. Observed clear zones were comparable for the oxidative stress tolerance, and protease activity suggesting no difference between strains.

The growth curves revealed distinct differences between the WT, Δ*aroA* mutant, and C*aroA* complemented strains. While the WT and C*aroA* strains exhibited similar growth patterns, the Δ*aroA* mutant showed a notable reduction in optical density, particularly at the start of the logarithmic phase. Despite this reduction, the Δ*aroA* mutant reached a final optical density comparable to that of the WT and C*aroA* strains, suggesting a temporary growth impairment (**Figure 11A**).

For the oxidative stress and protease activity, the *aroA* deletion mutant demonstrated an unchanged tolerance to H_2_O_2_ when compared to the wild-type and complemented strains, as observed by similar inhibition zones around the H_2_O_2_ disks. Additionally, protease activity assays showed no significant differences among the strains, suggesting that *aroA* deletion does not impair the strain’s ability to produce extracellular proteases (**Figures 11B, C**).

**Figure 12:**
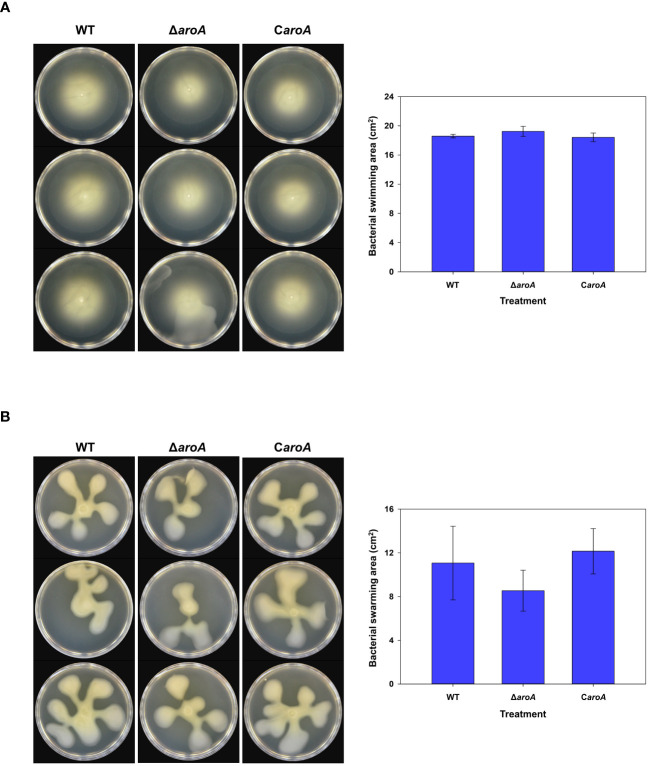
Comparative analysis of *Burkholderia plantarii* KACC 19864 wild-type, *aroA* deletion mutant, and *aroA*-complemented strains motility assays. **(A)** Swimming motility was assessed on 0.3% agar LB plates, and **(B)** swarming motility assay on 0.5% agar LB plates. After incubation, plates were imaged and the spread of bacterial growth was measured using ImageJ software. The bar graphs on the right side demonstrate the surface area of motility spread, with bars representing the mean from 3 replicates. Error bars represent standard deviation, illustrating no significant difference in motility among the strains.

For bacterial motility, after incubation, images captured from both swarming (**Figure 12A**) and swimming (**Figure 12B**) assays showed that the bacterial spread on the agar surfaces was comparable across all three strains. Quantitative analysis using ImageJ software confirmed that there were no statistically significant differences in the surface area of growth spread among the wild-type, *aroA* deletion mutant, and *aroA*-complemented strains (**Figure 12**).

## 4 Discussion

This study aimed to perform genome mining analysis of *B. plantarii* using all available complete and nearly complete genomes. In particular, the genome of *B. plantarii strain* KACC 18964 was sequenced in this study and compared with other available sequences. Whole-genome comparison using *B. glumae* BGR1 as a congeneric control indicated the taxonomic position of KACC 18964 as a *B. plantarii* strain. The genome of *B. plantarii* is relatively large, ranging from 7.90 to 8.08 Mb, and comprises two chromosomes and one plasmid. Notably, there was little variation in the genome size among the studied *B. plantarii* genomes, in contrast to the significant variation observed in the sizes of *B. glumae* genomes that have been previously reported. For example, *B. glumae* strain AU6208 exhibited the smallest genome size (approximately 4.9 Mbp), whereas the BGR1 strain had the largest genome size (approximately 7.2 Mbp). The relatively small genome size of *B. glumae* AU6208 may be attributed to genome rearrangements or deletions resulting from its adaptation to a different host.

In a previous comparative genomic study of phytopathogenic *Burkholderia* species, Seo et al. (2015) analyzed the genome sequences of different strains of *B. glumae*, *B. gladioli*, and *B. plantarii* and identified variations in genome size between species as well as among different strains of the same species. The large genome size of *B. plantarii* with multiple replicons indicates a versatile lifestyle that allows it to thrive in soil and infect plants. Larger genomes are thought to require more complex regulation of gene expression and an increased number of genes encoding regulatory proteins, and genome size and content are largely influenced by environmental pressures (Bentley and Parkhill, 2004; Mannaa et al., 2018).

The large *B. plantarii* genome harbors a diverse array of secondary metabolite gene clusters, as evidenced by the detection of 20 different secondary metabolite gene clusters using anti-SMASH analysis of the KACC 18964 genome. These metabolites likely confer various ecological benefits to *B. plantarii*, enabling their survival, interactions, and competition with the surrounding organisms. Among the detected clusters, 12 showed varying degrees of similarity to known gene clusters for secondary metabolites.

Notably, a cluster encoding non-ribosomal peptide metallophores and a non-ribosomal peptide synthetase displayed 100% similarity to a gene cluster encoding plantaribactin, a compound previously isolated and characterized from *B. plantarii* (DSM9505 = ATCC43733). This cluster has also been identified in other plant-associated *Burkholderia* and *Paraburkholderia* species (Hermenau et al., 2019). Plantaribactin is an iron-chelating and nitric oxide-donating diazeniumdiolate siderophore that, like other siderophores, is used by many bacterial pathogens to overcome iron limitations in the host environment. Therefore, plantaribactin may play a key role in conferring fitness benefits to microbes (Lamont et al., 2002; Johnson et al., 2013; Palmer and Skaar, 2016).

Additionally, another cluster with 100% similarity to a secondary metabolite gene cluster encoding a non-ribosomal peptide synthetase was detected, which was similar to the rhizomides found in *Paraburkholderia rhizoxinica*. Previous studies have demonstrated that rhizomes possess weak antibacterial properties, implying potential benefits for bacterial fitness (Wang et al., 2018). Furthermore, a gene cluster encoding a non-ribosomal peptide synthetase-like fragment was detected that exhibited 62% similarity to a cluster associated with fragin in *B. cenocepacia* (Jenul et al., 2018). While fragin is known for its antifungal properties and potential metal chelation activity, the similarity noted does not confirm the production of fragin or its related compounds in *B. plantarii*.

A cluster encoding a non-ribosomal peptide synthetase and type I polyketide synthase demonstrated 41% similarity to the siderophore yersiniabactin from *P. syringae* pv. Tomato (Jones et al., 2007). Another cluster encoding a non-ribosomal peptide synthetase, beta-lactone-containing protease inhibitor, and type I polyketide synthase showed 20% similarity to bacillomycin D from *Bacillus velezensis* (Koumoutsi et al., 2004). Bacillomycin D, which is produced by several *Bacillus* strains, has been shown to confer antagonistic activity against various microorganisms, including *Candida* spp., *Aspergillus flavus*, and *Fusarium graminearum* (Moyne et al., 2001; Olfa et al., 2015; Gu et al., 2017). Notably, several other secondary metabolite biosynthetic gene clusters showed low levels of similarity or no similarity to clusters of known metabolites, indicating that *B. plantarii* KACC 18964 could be a valuable source for studying novel bioactive secondary metabolites.

The secondary metabolite gene clusters identified in *B. plantarii* KACC 18964 genome not only demonstrate the bacterial potential for producing a wide spectrum of bioactive compounds but also reflect the intricate biological processes underlying its interaction with the environment. Particularly, the presence of diverse biosynthetic pathways, such as those for non-ribosomal peptides, polyketides, and terpenes, underscores the organism’s adaptability and underscores its potential to influence and thrive in various ecological niches (Raaijmakers and Mazzola, 2012). Future studies focusing on the expression, regulation, and functional analysis of these pathways can provide deeper insights into their roles in the organism’s life cycle and interactions, as well as their potential utility in various applications.

Another critical feature of *B. plantarii* is its ability to produce tropolone, a phytotoxin that represents the most significant virulence factor causing disease in rice (Azegami et al., 1987). Tropolone is a non-benzenoid aromatic compound that exhibits properties similar to those of phenols and acids. This compound possesses both antimicrobial and phytotoxic properties (Trust, 1975; Azegami et al., 1987). As a biotoxin, tropolone acts as a virulence factor for plant-pathogenic bacteria and displays varying degrees of toxicity towards several organisms. Consequently, its presence poses potential risks to agricultural production, plant-soil-water ecosystems, and human health (Liu et al., 2018).

Our results demonstrated the conservation of the operon consisting of three essential genes (*troR1, troK*, and *troR2*) responsible for regulation of tropolone biosynthesis across all examined *B. plantarii* genomes. Early experiments suggested that tropolone exposure led to disease manifestations in certain plants, including rice; however, others, such as *Gladiolus gandavensis*, remained symptom-free when treated solely with the phytotoxin. Notably, these gladiolus plants exhibited disease symptoms when inoculated with the toxin-producing bacterium *B. plantarii*. These findings imply that factors other than tropolone play significant roles in the virulence of *B. plantarii* (Iiyama et al., 1998).

The QS system, a critical cell density-dependent regulatory mechanism, is prevalent in various bacterial genera, including *Burkholderia*, and influences myriad bacterial biological processes and virulence characteristics (Mannaa et al., 2018). In *B. glumae*, the principal virulence factor, toxoflavin, is governed by the LysR family regulator, ToxR, which functions as a coinducer. Another regulatory element, ToxJ, plays a significant role in the regulation of the toxoflavin operon, and its expression is mediated by the TofI or TofR components of the QS system (Kim et al., 2004; Seo et al., 2015). The QS system regulates the transcription of specific genes at quorum concentrations in response to the accumulation of signal molecules in the surrounding medium. In gram-negative bacteria, *N*-acyl homoserine lactone (AHL) signaling molecules generated by an AHL synthase enzyme of the LuxI protein family are primarily used. AHL molecules bind to the transcriptional regulators of the LuxR family at quorum concentrations, thereby influencing the transcriptional regulation of target genes (Fuqua et al., 1994). This mechanism provides significant advantages to bacterial communities, particularly those that produce extracellular enzymes and virulence factors.

In *B. plantarii*, the QS system operon—comprising *plaI, plaM*, and *plaR* genes—closely resembles the corresponding system in *B. glumae*, as demonstrated by the analysis of four *B. plantarii* strains in which it was conserved. The AHL-QS mechanism in *B. plantarii* plays a pivotal role in the progression of rice seedling blight. Studies involving the generation of *plaR* knockout mutants highlight the significance of the AHL-QS regulatory system in the pathogenicity of *B. plantarii*, because disabling the *PlaIR* function delayed the onset of rice seedling blight significantly (Solis et al., 2006). Subsequent studies revealed that the application of AHL-QS inhibitors resulted in reduced tropolone production due to inhibited *plaI* expression (Wang et al., 2013). This discovery draws parallels between the regulation of toxoflavin in *B. glumae* and that of tropolone in *B. plantarii*, highlighting the importance of QS-related genes. However, the exact role of the QS system in *B. plantarii* requires further investigations.

Previously, the role of the CRISPR system was considered to be limited to safeguarding bacteria against invasion by bacteriophages or foreign plasmid DNA (Jansen et al., 2002). However, recent perspectives have expanded to include its broader ecological implications, such as its potential influence on virulence, biofilm formation in pathogenic bacteria, and as a barrier to horizontal gene transfer. Furthermore, research has revealed that environmental stimuli can modulate the expression of the CRISPR system and its role as a regulator of gene expression (Hatoum-Aslan and Marraffini, 2014; Louwen et al., 2014; Gao et al., 2015). Our findings indicated that the *B. plantarii* genome harbors near-complete CRISPR-Cas systems, unlike other rice-associated *Burkholderia* species. These systems encompassed a series of *Cas* genes that were conserved across the four examined *B. plantarii* genomes. We also observed high conservation levels within the direct repeats of the CRISPR systems across these genomes. These findings imply that the CRISPR systems in *B. plantarii* could serve distinct roles that subtly differentiate them from other closely related rice-pathogenic bacteria (*i.e.*, *B. glumae* and *B. gladioli*) despite their similarities (Seo et al., 2015).

In strains ATCC 43733 and KCCM 18964, we identified two CRISPR systems, whereas PG1 and ZJ171 had three. The sequence of CRISPR 3 was consistent between PG1 and ZJ171. A previous study on *B. plantarii* PG1 detected three CRISPR arrays, indicating their potential importance in plant interactions (Gao et al., 2015), and reported the QS-dependent regulation of CRISPR/Cas genes, which may affect the bacterial immune system in *B. plantarii* PG1. The detection of nearly complete CRISPR systems in *B. plantarii* genomes emphasizes the necessity for further exploration of their possible functional roles.

The analysis of genomic islands across the examined genomes uncovered a variety of consistent GIs among different strains. Detailed study of *B. plantarii* KACC 18964 as representative genome revealed the prediction of 59 unique genomic islands, rich in genes of uncharacterized function and mobile genetic elements, indicates the complex genetic architecture that may fortify the ecological fitness and pathogenic potential of the bacterium (Juhas et al., 2009; Langille and Brinkman, 2010). These GIs likely function as reservoirs for virulence-related genes, underscoring the bacterial ability to adapt and interact with plant hosts.

The identification of specific virulence and fitness-related genes within these islands, such as toxin family proteins and type VI secretion system effector proteins, aligns with previous studies underscoring the role of such genes in bacterial competitiveness and host interaction (Russell et al., 2011; Hood et al., 2010). These genetic elements may contribute to the bacterial ability to affect rice plant health directly through pathogenic mechanisms or indirectly through competitive suppression of beneficial microbiota.

The presence of mobile genetic elements and toxin-antitoxin systems suggests a genetic toolkit for rapid adaptation and survival under selective pressures, such as plant immune responses or microbial competition (Hacker and Carniel, 2001).

Further research should investigate the functional roles of these GIs, particularly their gene expression in response to environmental stimuli and their influence on the host-pathogen interface.

The ability of gram-negative bacteria, including *Burkholderia* species, to regulate the translocation of powerful effectors from their synthesis sites to the cellular exterior or even to neighboring organisms is a crucial survival and communication mechanism (Green and Mecsas, 2016). *Burkholderia* spp., as demonstrated by genomic studies, exhibit diverse secretion systems, each potentially playing a key role in virulence, communication, and competitive interaction with other microbes (Seo et al., 2015). The present study indicates that the four *B. plantarii* genomes have various types of secretion systems, including T1SS, T2SS, and T3SS; several clusters of T4SS, T5SS, and T6SS; and associated genes. Different types of secretion systems can serve unique functions, but different clusters of a particular secretion system may also play distinctive roles. Kim et al. (2020) reported such functional distinctions among T6SS clusters in the related species *B. glumae*; one cluster was associated with virulence in rice plants, whereas the other was connected to bacterial competition within the surrounding microbial community. These traits are essential for *B. glumae* to compete effectively, establish a population, and induce disease in rice.

Among the various secretion systems in gram-negative bacteria, the T3SS serves as an effective molecular syringe, enabling gram-negative bacteria to penetrate, grow, and survive in eukaryotic hosts. Numerous animal and plant pathogens utilize T3SS to inject a suite of proteins, known as bacterial ‘effector proteins,’ into the cytosol of host cells. These proteins can modulate eukaryotic regulatory or signaling pathways during bacterial infection, causing a range of symptoms (Puhar and Sansonetti, 2014; Radics et al., 2014). Therefore, our study aimed to examine the potential involvement of the T3SS in *B. plantarii* virulence in rice by investigating the potential effector proteins. T3SS was conserved across the genomes of the four *B. plantarii* strains examined. *In silico* analysis using different algorithms for potential effectors yielded a large number of potential effectors. To prioritize the most promising candidates, we focused on the *aroA* gene, a potential effector that was detected by all algorithms in the four studied *B. plantarii* genomes.

The *aroA* gene, which is ubiquitous in many bacterial species, encodes the enzyme 3-phosphoshikimate 1-carboxyvinyltransferase, which is a crucial participant in the shikimate pathway. This pathway is responsible for aromatic amino acid synthesis (Duncan et al., 1984). Notably, the shikimate pathway is present in plants and various microorganisms but is absent in mammals, making it an ideal target for herbicidal strategies. For instance, glyphosate (commercially known as Roundup), a common herbicide, functions by inhibiting the synthase enzyme, disrupting the production of essential amino acids in plants and consequently leading to their demise (Funke et al., 2006).

Our study revealed that an *aroA* deletion mutant of *B. plantarii* KACC 18964 significantly reduced bacterial virulence in rice plants. This finding is consistent with previous research, where deletion of the *aroA* gene in *B. glumae* similarly impaired its virulence in rice (Karki and Ham, 2014). However, it is essential to clarify the exact role of *aroA*, identified here as a potential effector of T3SS. We are currently investigating whether the reduced virulence in *aroA* deletion mutant, results from changes in the bacterial ability to produce certain metabolites or whether it can be attributed to the role of *aroA* as a T3SS-mediated effector. The unchanged oxidative stress response, protease activity and motility in the *ΔaroA* mutant alongside the temporary growth delay suggest that *aroA* plays a non-essential role in these specific traits. The ability to withstand oxidative stress is a vital trait for pathogens, allowing them to survive the burst of reactive oxygen species produced as a part of the plant’s innate immune response (O’Brien et al., 2012). Similarly, extracellular proteases play a critical role in phytopathogen virulence by degrading plant structural proteins, facilitating bacterial invasion, and disabling plant defense proteins (Figaj et al., 2019). The absence of significant changes in protease activity and H_2_O_2_ tolerance suggests that *aroA* role in virulence may not directly be linked to these mechanisms but could instead be related to pathogenic interactions within the plant host. However, the observed growth pattern disruption indicates its potential involvement in bacterial metabolism. This supports the hypothesis that *aroA* could be serving dual functions: as an enzyme within the shikimate pathway and, potentially, as a T3SS effector that interferes with plant metabolism through molecular mimicry. This dual functionality hypothesis could be supported by the understanding that T3SS effectors often mimic eukaryotic proteins to facilitate pathogenicity, coupled with the fact that the shikimate pathway, crucial for the synthesis of aromatic amino acids, is present in both plants and bacteria. The conservation of this pathway across different biological kingdoms provides a molecular basis for *aroA* multifunctionality, enabling it to participate in bacterial metabolism and, when introduced into plant cells, to potentially interfere with analogous metabolic processes through molecular mimicry (Coburn et al., 2007; Dean, 2011; Shende et al., 2024). The *aroA* product may have evolved to acquire an entirely new function as a virulence factor when secreted into host cells. These fundamental questions about *aroA* function in *B. plantarii* pathogenesis remain to be explored. Further investigation is crucial to substantiate these hypotheses and to elucidate the precise roles of *aroA*, particularly in validating its bifunctional nature—operating both within the bacterium and within the host plant as a T3SS effector.

In conclusion, this comprehensive study offers novel insights into the genomic features, virulence, and ecological adaptability of the rice pathogenic *B. plantarii*. Through meticulous genomic analysis, including comparison among *B. plantarii* genomes and identification of unique secondary metabolite gene clusters, our research elucidates the complex genetic make-up that potentially underlies *B. plantarii* pathogenicity and environmental interactions. The detailed investigation of CRISPR-Cas systems and genomic islands further accentuates the genetic diversity and evolutionary potential of *B. plantarii*, highlighting its adaptability and the dynamic nature of its interactions within diverse environments. Our findings not only contribute to the understanding of *B. plantarii* genomic complexity and pathogenic mechanisms but also open avenues for future research focused on exploring the functional roles of identified gene clusters and *aroA*, as a potential T3SS effector.

## Data availability statement

The original contributions presented in the study are included in the article/**Supplementary Material**. Further inquiries can be directed to the corresponding author.

## Author contributions

MM: Conceptualization, Data curation, Formal analysis, Investigation, Writing – original draft, Writing – review & editing. DL: Conceptualization, Data curation, Methodology, Writing – original draft. H-HL: Conceptualization, Data curation, Methodology, Software, Writing – original draft. GH: Data curation, Formal analysis, Methodology, Writing – review & editing. MK: Data curation, Formal analysis, Methodology, Writing – review & editing. T-JK: Visualization, Writing – review & editing. JP: Formal analysis, Writing – review & editing. Y-SS: Conceptualization, Funding acquisition, Investigation, Project administration, Resources, Supervision, Validation, Visualization, Writing – review & editing.
