# Supplementary material for: Exploring the comparative genome of rice pathogen Burkholderia plantarii: unveiling virulence, fitness traits, and a potential type III secretion system effector
Source: Front Plant Sci. 2024 May 23;15:1416253. doi: 10.3389/fpls.2024.1416253 (PMC11153758; doi:10.3389/fpls.2024.1416253)
Supplement: Supplementary file 1 [file Table_1.docx]

Supplementary Material

**Supplementary Table 1.**

Putative secondary metabolites gene clusters detected using antiSMASH version 7, showing the type, position of the genome, most similar known clusters, and similarity percentage for *Burkholderia plantarii* KACC 18964

| contig1 | Region | Type | From | To | Most similar known cluster | Similarity (%) | Reference |
| --- | --- | --- | --- | --- | --- | --- | --- |
|  | 1 | Type I PKS (Polyketide synthase), Non-ribosomal peptide synthetase-like fragment | 202,804 | 248,332 | _ |  |  |
|  | 2 | Terpene | 689,150 | 709,983 | _ |  |  |
|  | 3 | Non-ribosomal peptide synthetase | 918,170 | 960,693 | _ |  |  |
|  | 4 | Non-ribosomal peptide synthetase-like fragment | 1,034,457 | 1,075,123 | [Pyoverdine DC3000](https://mibig.secondarymetabolites.org/go/BGC0002571/1)  (*Pseudomonas syringae pv. tomato*) | 3 | (Ravel & Cornelis, 2003) |
|  | 5 | Type I PKS (Polyketide synthase) | 2,984,832 | 3,032,433 | [capsular polysaccharide (](https://mibig.secondarymetabolites.org/go/BGC0000736/1)*[Mannheimia](https://mibig.secondarymetabolites.org/go/BGC0000736/1)**[haemolytica](https://mibig.secondarymetabolites.org/go/BGC0000736/1)*[)](https://mibig.secondarymetabolites.org/go/BGC0000736/1) | 25 | (Lo et al., 2001) |
|  | 6 | Homoserine lactone | 3,150,433 | 3,171,011 | _ |  |  |
|  | 7 | Non-ribosomal peptide synthetase | 3,610,298 | 3,667,373 | _ |  |  |
| contig2 |  |  |  |  | _ |  |  |
|  | 8 | Phosphonate | 50267 | 64931 | [Phosphinothricintripeptide (](https://mibig.secondarymetabolites.org/go/BGC0000406/1)*[Streptomyces viridochromogenes](https://mibig.secondarymetabolites.org/go/BGC0000406/1)*[)](https://mibig.secondarymetabolites.org/go/BGC0000406/1) | 6 | (Schwartz et al., 2004) |
|  | 9 | Non-ribosomal peptide synthetase, Type I PKS (Polyketide synthase) | 583313 | 636467 | [Yersiniabactin (](https://mibig.secondarymetabolites.org/go/BGC0002570/1)*[Pseudomonas syringae pv. tomato](https://mibig.secondarymetabolites.org/go/BGC0002570/1)*[)](https://mibig.secondarymetabolites.org/go/BGC0002570/1) | 41 | (Jones et al., 2007) |
|  | 10 | Non-ribosomal peptide synthetase-like fragment, Beta-lactone containing protease inhibitor | 647516 | 689419 | [Fragin (*Burkholderia cenocepacia*)](https://mibig.secondarymetabolites.org/go/BGC0001599/1) | 62 | (Jenul et al., 2018) |
|  | 11 | Non-ribosomal peptide metallophores, Non-ribosomal peptide synthetase | 711967 | 796373 | [Plantaribactin (*Burkholderia plantarii*)](https://mibig.secondarymetabolites.org/go/BGC0002565/1) | 100 | (Hermenau et al., 2019) |
|  | 12 | Non-ribosomal peptide synthetase | 822858 | 885348 | [Rhizomide A/rhizomide B/rhizomide C (](https://mibig.secondarymetabolites.org/go/BGC0001758/1)*[Paraburkholderia rhizoxinica](https://mibig.secondarymetabolites.org/go/BGC0001758/1)*[)](https://mibig.secondarymetabolites.org/go/BGC0001758/1) | 100 | (Wang et al., 2018) |
|  | 13 | β-lactam | 2026747 | 2048237 | [1-carbapen-2-em-3-carboxylic acid (](https://mibig.secondarymetabolites.org/go/BGC0001847/1)*[Pectobacterium carotovorum](https://mibig.secondarymetabolites.org/go/BGC0001847/1)*[)](https://mibig.secondarymetabolites.org/go/BGC0001847/1) | 16 | (McGowan et al., 1997) |
|  | 14 | [Terpene](https://docs.antismash.secondarymetabolites.org/glossary/#terpene) | 2121746 | 2138385 | _ |  |  |
|  | 15 | Redox-cofactors such as PQQ (NC_021985:1458906-1494876) | 2206608 | 2228842 | [Lankacidin C (](https://mibig.secondarymetabolites.org/go/BGC0001100/1)*[Streptomyces rochei](https://mibig.secondarymetabolites.org/go/BGC0001100/1)*[)](https://mibig.secondarymetabolites.org/go/BGC0001100/1) | 13 | (Suwa et al., 2000) |
|  | 16 | [Terpene](https://docs.antismash.secondarymetabolites.org/glossary/#terpene) | 2266757 | 2288594 | _ |  |  |
|  | 17 | Non-ribosomal peptide synthetase, Beta-lactone containing protease inhibitor, Type I PKS (Polyketide synthase) | 2365655 | 2432143 | [Bacillomycin D (*Bacillus velezensis*)](https://mibig.secondarymetabolites.org/go/BGC0001090/1) | 20 | (Koumoutsi et al., 2004) |
|  | 18 | [Beta-lactone containing protease inhibitor, Terpene](https://docs.antismash.secondarymetabolites.org/glossary/#terpene) | 2563979 | 2602709 | [Barbamide (](https://mibig.secondarymetabolites.org/go/BGC0000962/1)*[Lyngbya majuscula](https://mibig.secondarymetabolites.org/go/BGC0000962/1)*[)](https://mibig.secondarymetabolites.org/go/BGC0000962/1) | 33 | (Chang et al., 2002) |
|  | 19 | Non-ribosomal peptide synthetase, Homoserine lactone | 2749880 | 2796386 | [Bactobolin (](https://mibig.secondarymetabolites.org/go/BGC0000961/1)*[Burkholderia thailandensis](https://mibig.secondarymetabolites.org/go/BGC0000961/1)*[)](https://mibig.secondarymetabolites.org/go/BGC0000961/1) | 9 | (Carr et al., 2011) |
|  | 20 | Homoserine lactone | 3554445 | 3575056 | __ |  |  |

**Supplementary Table 2.**

Primers used in this study

| Primer | Role | Sequence |
| --- | --- | --- |
| aroA_LF | Amplifying L fragment | 5'- TTTCCCGGGACGAGCTCGACGCCTACA -3' |
| aroA_LR | Amplifying L fragment | 5'- GTGACGACGAGATAGTCGGCATGGGAGTAGGGACCGA -3' |
| aroA_RF | Amplifying R fragment | 5'- ATGTCGGTCCCTACTCCCATGCCGACTATCTCGTCGT -3' |
| aroA_RR | Amplifying R fragment | 5'- TTTGGATCCTGGAATGGTCGAGTCGATTT -3' |
| aroA_UP_F | Confirming for mutants | 5'- GCGGAGCTGAAATTTTCGT -3' |
| aroA_UP_R | Confirming for mutants | 5'- CTGTCGAGCAGGTGAAAGC -3' |

**Supplementary Table 3.**

Bacterial strains and plasmids used in this study

| Bacterial strain | Role | Reference |
| --- | --- | --- |
| *B. plantarii* |  |  |
| KACC 18964 | Wild-type | (Ra et al., 2016) |
| *ΔaroA* | KACC 18964 *ΔaroA* | This study |
| C*aroA* | KACC18964 harboring *ΔaroA,* pBBR1 MCS-2 P2-3 *aroA* | This study |
|  |  |  |
| *E. coli* |  |  |
| DH5α λpir | For cloning and replication of plasmids | Lab collection |
| S17-1 λpir | For transferring cloning plasmids to recipient cells | Lab collection |
|  |  |  |
| Plasmids |  |  |
| pK18mobsacB | Allelic exchange suicide vector, sacB, Km | (Schäfer et al., 1994) |
| pBBR1 MCS-2 P2-3 | Broad host range overexpression vector, KM | (Kovach et al. 1995) |
| pBBR1 MCS-2 P2-3 aroA | pBBR1 MCS-2 P2-3 vector including *aroA* gene | This study |
